# T Cell Transcriptomes Are Dynamically Modulated by Microgravity In Vivo Along Core Gene Networks and Key Molecular Pathways

**DOI:** 10.3390/cells15212017

**Published:** 2026-11-05

**Authors:** Daria D. Vlasova, Anastasia A. Kotikova, Viacheslav A. Shmarov, Tatiana A. Shigueva, Ilya V. Rukavishnikov, Elena S. Tomilovskaya, Milos Ljubisavljevic, Dhifaf Sarhan, Nik V. Kuznetsov

**Affiliations:** 1 Institute of Biomedical Problems, Russian Academy of Sciences (IBMP RAS), Moscow 123007, Russiashmarov.v.a@gmail.com (V.A.S.); t.shigueva@gmail.com (T.A.S.); finegold@yandex.ru (E.S.T.); 2 Chair of Biochemistry and Molecular Biology, Pirogov Russian National Research Medical University, Moscow 117513, Russia; 3 Department of Physiology, College of Medicine and Health Sciences, United Arab Emirates University, Al Ain P.O. Box 15551, United Arab Emirates; 4 ASPIRE Precision Medicine Research Institute Abu Dhabi, United Arab Emirates University, Al Ain P.O. Box 15551, United Arab Emirates; 5 Department of Laboratory Medicine, Division of Pathology, Karolinska Institute, SE-141 86 Stockholm, Sweden; 6 Karolinska Institute, Nobels väg 16, SE-171 77 Stockholm, Sweden

**Keywords:** T cell, gene expression, transcriptome, microgravity, pathway, gene network

## Abstract

**Highlights:**

**What are the main findings?**

- In a unique ground-based study, three weeks of simulated microgravity involving ten healthy volunteers was sufficient to induce widespread deregulation of core T cell transcriptional programs in vivo, affecting gene networks involved in cell proliferation, cell-cycle regulation, and genome integrity.
- T cell transcriptomic responses to microgravity were dynamic and time-dependent, peaking during mid-to-late exposure and showing partial recovery afterward, with substantial inter-individual variability in response.

**What are the implications of the main findings?**

- Microgravity alone, even at levels causing no detectable systemic effects, is sufficient to perturb the equilibrium of gene expression and regulatory networks critical for immune function and cellular health during spaceflight.
- The pronounced individual heterogeneity in T cell responses suggests that effective countermeasures for astronauts may require personalized medicine approaches rather than universal solutions, with implications for biomarker development and tailored interventions in future long-duration missions.

**Abstract:**

**Background**: The health of astronauts and success of space missions are contingent on resilience to the cumulative stressors of the space environment, including radiation, isolation, confinement and microgravity. While these factors are known to disrupt physiological function at molecular and cellular levels, the isolated impact of microgravity itself remains poorly defined. **Objective**: To explore in vivo effects of long-term Simulated MicroGravity (SMG) on T cell transcriptomes by analyzing gene expression changes in peripheral blood T cells of healthy volunteers. **Methods**: A three-week SMG exposure was completed by ten healthy male participants, during which peripheral blood specimens were harvested at five critical junctures (covering baseline, intra-exposure and recovery stages). Post-collection, T lymphocytes were enriched, and their total RNA was extracted. Transcriptome analysis was performed by RNA-seq, the gold standard for transcriptomics owing to its unbiased approach to gene detection and quantification, which provides genome-wide quantification with global normalization and, through a genome-wide analytical approach, the statistical framework to detect differentially expressed genes with controlled error rates. Following next-generation sequencing, a rigorous bioinformatics framework was employed to analyze and interpret the volunteers’ global gene expression dynamics. **Results**: Ground-based SMG induces widespread deregulation of gene-regulatory circuitry within T lymphocytes. SMG is associated with transcriptome shifts in several key molecular pathways in vivo including those involved in cell proliferation, cell cycle regulation, maintenance of genome integrity, as well as oncogenic and tumor suppressor gene networks. Corresponding hub genes were differentially expressed in the majority of tested healthy volunteers. **Conclusions**: SMG perturbs essential intracellular gene-regulatory networks in human T cells in vivo. The mechanical unloading imposed by microgravity, even in the absence of detectable systemic effects, was sufficient to deregulate core gene networks, upset the transcript-level equilibrium of central molecular pathways and alter associated gene expression profiles.

## 1. Introduction

The spaceflight environment presents a complex spectrum of physiological stressors that profoundly differ from conditions on Earth, posing significant risks to astronaut health and mission success. NASA has categorized these into the following five primary hazards: isolation, confinement, distance from Earth, gravity alterations, and exposure to space radiation [1,2]. Furthermore, additional factors such as hypomagnetic environment, circadian rhythm disruption, and perturbations in the environmental microbiome can further compound these challenges and influence crew wellbeing [3,4]. Among these stressors, the effects of galactic cosmic radiation (GCR) on biomolecular and cellular processes have been extensively characterized through both spaceflight and ground-based simulation studies [5,6]. In contrast, the specific contribution of microgravity to cellular dysfunction, particularly in immune cell signaling, proliferation, and metabolism, remains insufficiently defined and a subject of active investigation. Recent evidence suggests that MG may be a dominant driver of the physiological stress observed in spaceflight, significantly impacting biological systems [7,8]. To delineate the specific in vivo impacts of microgravity on human T lymphocytes, we applied long-term SMG to healthy volunteers [9]. RNA-seq is the gold standard for transcriptomics, since it provides an unbiased approach to gene detection and quantification. Our analysis of T cell transcriptomes revealed significant deregulation of core gene networks and key molecular pathways. A critical and novel finding was the observation of substantial inter-individual heterogeneity, with several distinct types of transcriptional responses identified among the volunteers. This evidence compellingly supports a shift in perspective: rather than pursuing a universal solution, effective countermeasures may require a personalized medicine approach that accounts for individual variability in response to microgravity. A microgravity investigation, conducted on a cohort of ten healthy individuals, was designed to determine whether the absence of gravity alone elicits detectable changes in T cell transcriptomes in vivo. Our findings could not only shed light on fundamental mechanisms of space cell biology but also serve as a pivotal stepping stone toward developing targeted strategies to safeguard human health during extended stays in microgravity.

## 2. Methods

### 2.1. Simulated Microgravity Experiment Setup

Ten healthy male volunteers of East Slavic ethnicity, aged 24 to 32 years, participated in a three-week simulated microgravity (SMG) experiment using dry immersion, as outlined in [7,10]. The dry immersion protocol was conducted at IBMP RAS, and the study was approved by the Biomedical Ethics Committee of the IBMP RAS and Section of Physiology of the Bioethics Committee of the UNESCO National Bioethics Commission approved this study (meeting no. 483 took place on 3 August 2018). All volunteers gave their written consent prior to the study, adhering to the ethical requirements of the Helsinki Code of Medical Ethics regarding Human Specimens.

Inclusion criteria were: medically and psychologically healthy males aged 21–40 years, height ≤ 180 cm, BMI 19–30 kg/m^2^, available for >5 weeks (including the 21-day exposure), and successfully medically cleared. Exclusion criteria were: any addiction (alcohol or drugs), smoking, chronic disease or recent surgery, acute bacterial or nonbacterial inflammatory disease (including COVID-19 positivity within 1 week to 24 h before the start date), regular medication use, blood donation >350 mL within 3 months, or participation in another study within the previous year [10]. No laboratory testing for viral infections or viral carrier status (e.g., EBV, CMV, and HPV) was performed.

During the SMG period, volunteers remained continuously in a dry immersion bath for 21 days without interruption; physical activity was restricted, and no physical or pharmacological countermeasures were administered. Volunteers were medically monitored throughout the study. As reported in [10], no clinical symptoms were observed or reported by any of the ten volunteers: no nausea, fever, or other signs of health deterioration were noted, and body temperature remained within the normal range. Mean daily body temperature during immersion (36.36 ± 0.29 °C) was slightly lower than at baseline (36.54 ± 0.29 °C). Transient back pain, predominantly in the lumbar region, was reported during the first 2 to 3 days of exposure and resolved by day 4, with no further complaints thereafter.

### 2.2. Peripheral Blood Sample Collection

**Figure 1:**
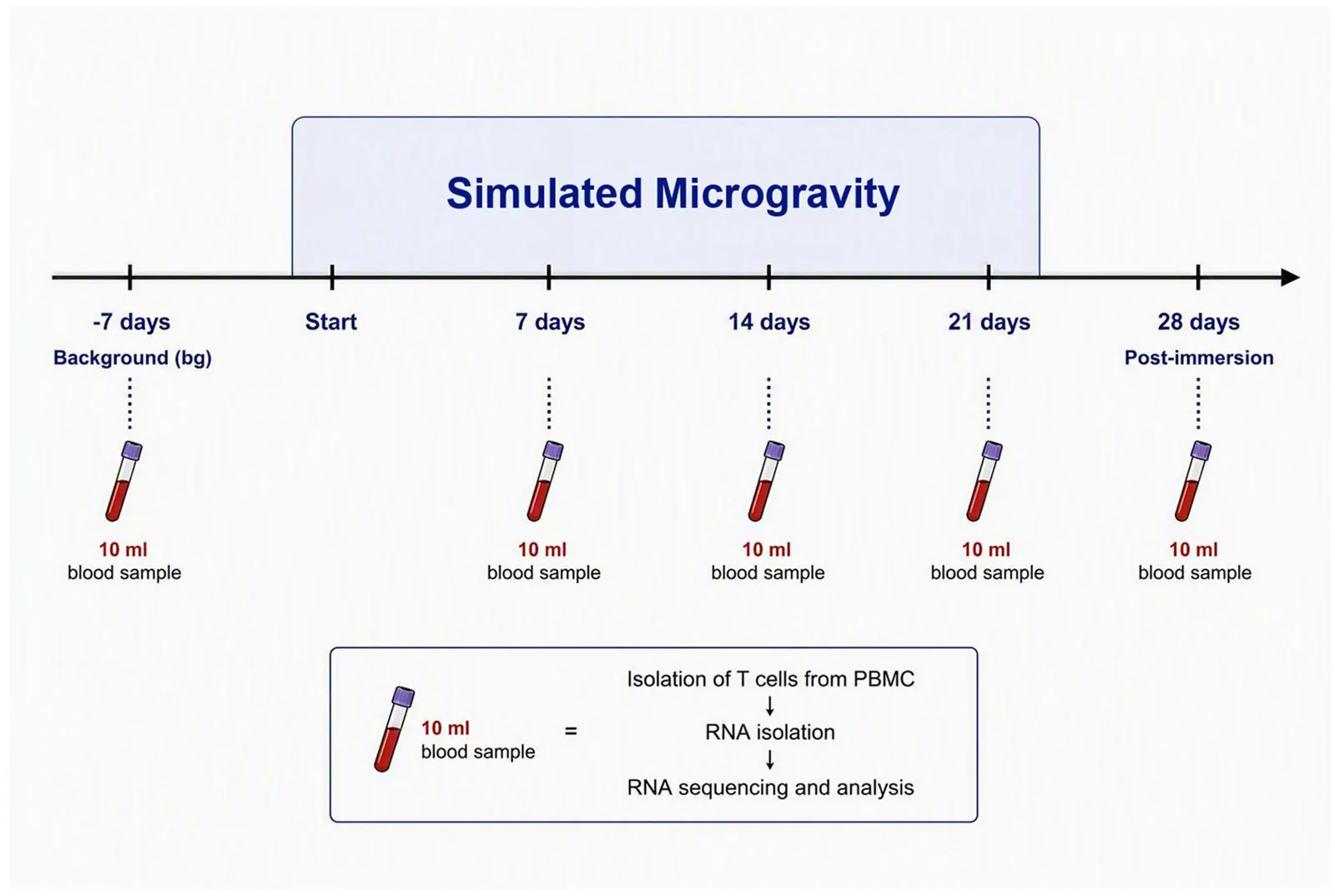
**Procedural setup for the ground-based SMG study.** The time course of the SMG experiment and sample processing flow chart.

During the SMG protocol, peripheral blood samples (10 mL each) were collected from every volunteer into sodium heparin tubes at the following five predefined time points: 7 days prior to SMG onset (day −7; background, bg), at 7, 14, and 21 days of microgravity exposure, and 7 days after the end of SMG exposure (day 28) (Figure 1). Volunteers were therefore under dry immersion from day 1 to day 21; exposure ended before the day 28 sampling. The number and spacing of sampling time points were constrained by the maximum blood volume permitted per volunteer under the study’s ethical approval; within these constraints, the immersion period was divided into equal weekly intervals, and the day −7 and day 28 samples provided pre-immersion reference values and an assessment of post-immersion recovery, respectively.

### 2.3. CD3+ T Cell Isolation

PBMCs were isolated by density gradient centrifugation using Ficoll-Paque™ PLUS (Cytiva, Uppsala, Sweden) according to the manufacturer’s instructions. T cells were enriched by positive CD3 selection using the EasySep™ Human CD3 Positive Selection Kit II (STEMCELL Technologies, Vancouver, BC, Canada). Purity was assessed by flow cytometry on representative samples and was approximately 95%, broadly consistent with the manufacturer’s specification of up to 99%. Although the remaining fraction may have comprised other cell populations whose highly expressed transcripts could contribute signal, an identical isolation protocol was applied to all samples and time points.

### 2.4. RNA Extraction and Sequencing

RNA was extracted, quantified, and RNA quality was assessed via AGE in 1% UltraPure Agarose (Cat. #16500500; Invitrogen, Waltham, MA, USA). Gel imaging conducted on ImageQuant LAS 4000 imaging system. Following successful completion of both internal and external quality control assessments (Novogene, Hong Kong), the RNA libraries were processed to 150 bp paired-end PE sequencing (Illumina HiSeq; Novogene), followed by standard data quality control procedures.

### 2.5. RNA-Seq Bioinformatics

#### 2.5.1. Genome Mapping and Transcript-Level Quantification Software

Sequencing reads were mapped to the human reference genome assembly GRCh38/hg38 (December 2013) using TopHat v2.0.12 with a maximum of two mismatches. Transcript-level quantification was performed with HTSeq v0.6.1 using the union mode. The quality of the resulting RNA-seq data was assessed, and key metrics are summarized in Supplementary File S7 (Table S1). All bioinformatic software applications and their versions are listed in Supplementary File S8 (Table S2), and the differential expression parameters are detailed in Supplementary File S8 (Table S3).

#### 2.5.2. Differential Expression Analysis

Because the study design provides a single sample per time point per volunteer, differential expression was assessed using DEGseq v1.12.0, which is specifically designed for comparisons between samples without biological replicates [11]. DEGseq models read counts using a negative binomial distribution, *K*_ij_~NB(μ_ij_, σ_ij_^2^), with a Poisson distribution *K*_ij_~P(μ_ij_) as a special case, and estimates dispersion by borrowing information across genes. For each volunteer separately, transcript levels at each post-baseline time point (days 7, 14, and 21 of exposure, and day 28 recovery) were compared with that volunteer’s own pre-immersion baseline (day −7). By design, this per-subject comparison controls for individual baseline differences: each volunteer serves as their own reference, and between-subject variance, which the PCA showed to dominate the dataset, is removed entirely rather than modeled as a covariate. Normalization was performed using the TMM method. Genes with an absolute log2 fold change > 1 and an adjusted *p*-value (Padj) < 0.005 after Benjamini–Hochberg correction were considered significantly differentially expressed.

#### 2.5.3. Pattern Identification and Visualization

**Figure 2:**
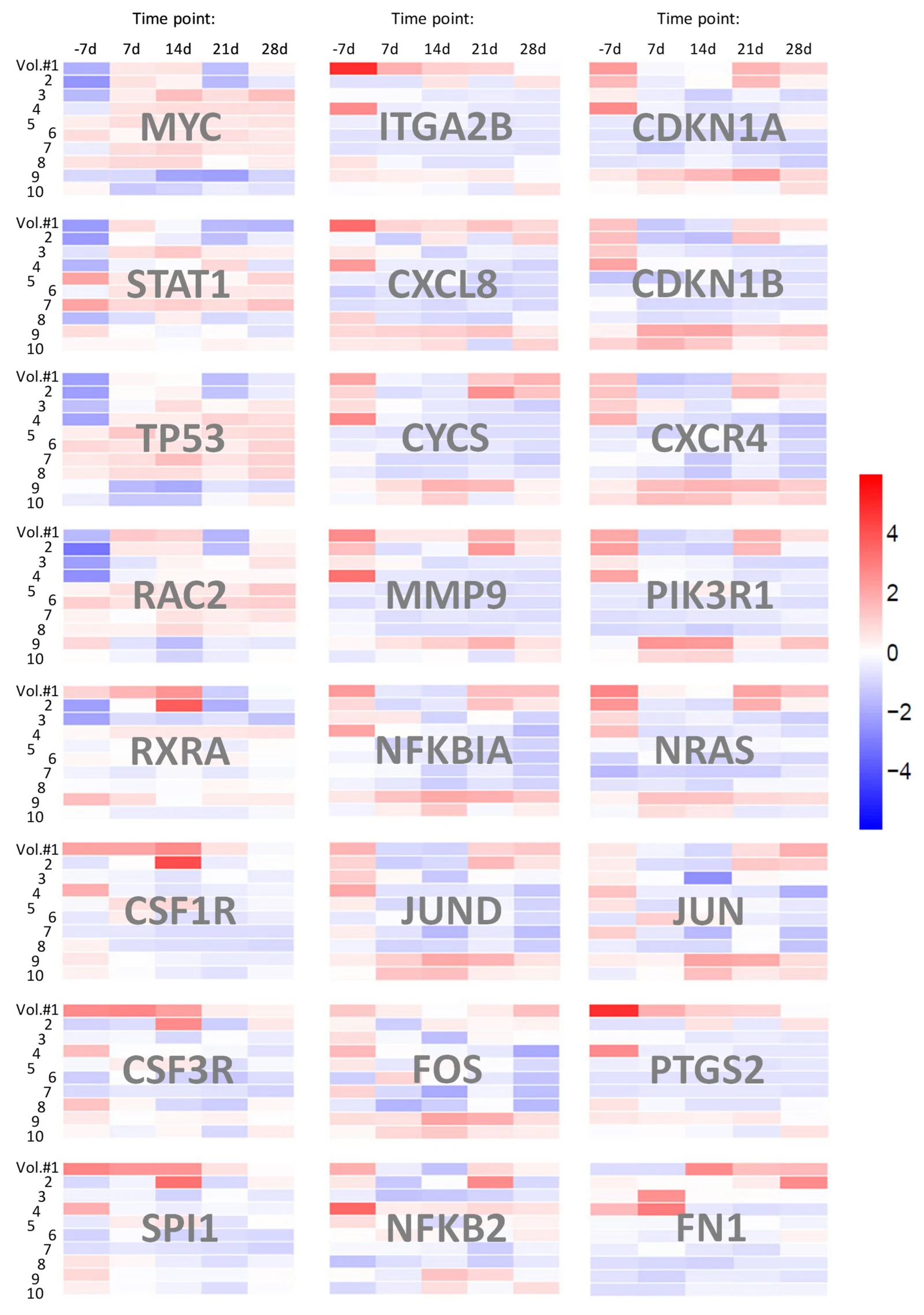
**Expression patterns of 24 hub genes from the KEGG Pathways in cancer (hsa05200) across the SMG time course.** Expression values for each of the 24 hub DEGs from hsa05200 were analyzed for each of the 10 volunteers across five time points (day −7 baseline, days 7, 14, and 21 of exposure, and day 28 recovery) and are shown as 24 heatmap panels, with volunteer number (Vol. #1–10) on the vertical axis and time points on the horizontal axis. Color scale indicates log2 fold change, with red indicating upregulation and blue indicating downregulation.

Expression patterns were identified by clustering log2-transformed ratios using hierarchical clustering (H-cluster), K-means partitioning, and self-organizing maps (SOMs). PCA and heatmaps were generated in NASQAR v1.0 (NYUAD) using the log2 normalized count matrix. Both normalized expression values (Supplementary File S1) and non-normalized expression values (Figure 2; Supplementary File S6) were used in the analysis of differentially expressed genes, permitting participant stratification as well as individual-level comparison.

#### 2.5.4. Functional Annotation and Enrichment

Functional classification of the refined data for peak-related genes was performed using the NCBI Gene resource as described previously [7,12]. GO enrichment was performed using GOSeq, topGO, and hmmscan v2.12, with a corrected *p*-value threshold of <0.05. KEGG pathway enrichment was performed using KOBAS v3.0 with a corrected *p*-value threshold of <0.05. Hub genes were defined as DEGs present in at least two of the top twelve most statistically significant KEGG pathways (Supplementary File S3).

## 3. Results

### 3.1. RNA-Seq and Gene Expression Data

A total of fifty samples (10 volunteers × 5 time points) yielded up to 58,676 transcripts expression data, which were assigned to over 200 biomolecular pathways. RNA-seq dataset is publicly accessible via the NCBI GEO database under accession number GSE301964.

Differential expression analysis was performed for T cell samples at each time point, with background expression values derived from the pre-immersion baseline (−7 d). Transcript levels of differentially expressed genes (DEGs) were visualized on volcano plots of −log10 (q-value) vs. log2(FC) for each time point (Supplementary File S1).

### 3.2. Gene Networks and Pathways

To identify the most prominently affected cell signaling networks, twelve KEGG pathways with the highest statistical significance (lowest corrected *p*-values) were selected from the complete pathway annotation list (Table 1; Supplementary File S2).

From the DEG mapping to these twelve top-ranked pathways, 44 interconnected genes that appeared in at least two of the selected pathways were designated as hub genes (Supplementary File S3). These genes (Table 2) were subsequently subjected to detailed comparative expression analysis across all time points (Figure 2; Supplementary File S6).

### 3.3. Cancer Genes’ Expression Patterns and Oncogenic Pathways

A panel of DEGs was mapped to the following three cancer-related KEGG pathways that were among the top twelve most statistically significant pathways: hsa05200, hsa05206, and hsa05202.

Among the 34 detected DEGs mapping to the KEGG Pathways in cancer (hsa05200), 24 genes qualified as hub genes based on their presence in at least two of the top twelve pathways (Supplementary File S4). Non-normalized expression values for each of the 24 hub genes were analyzed individually for each of the 10 volunteers across all time points (Figure 2) with the aligned expression patterns.

### 3.4. Gene Co-Expression

**Figure 3:**
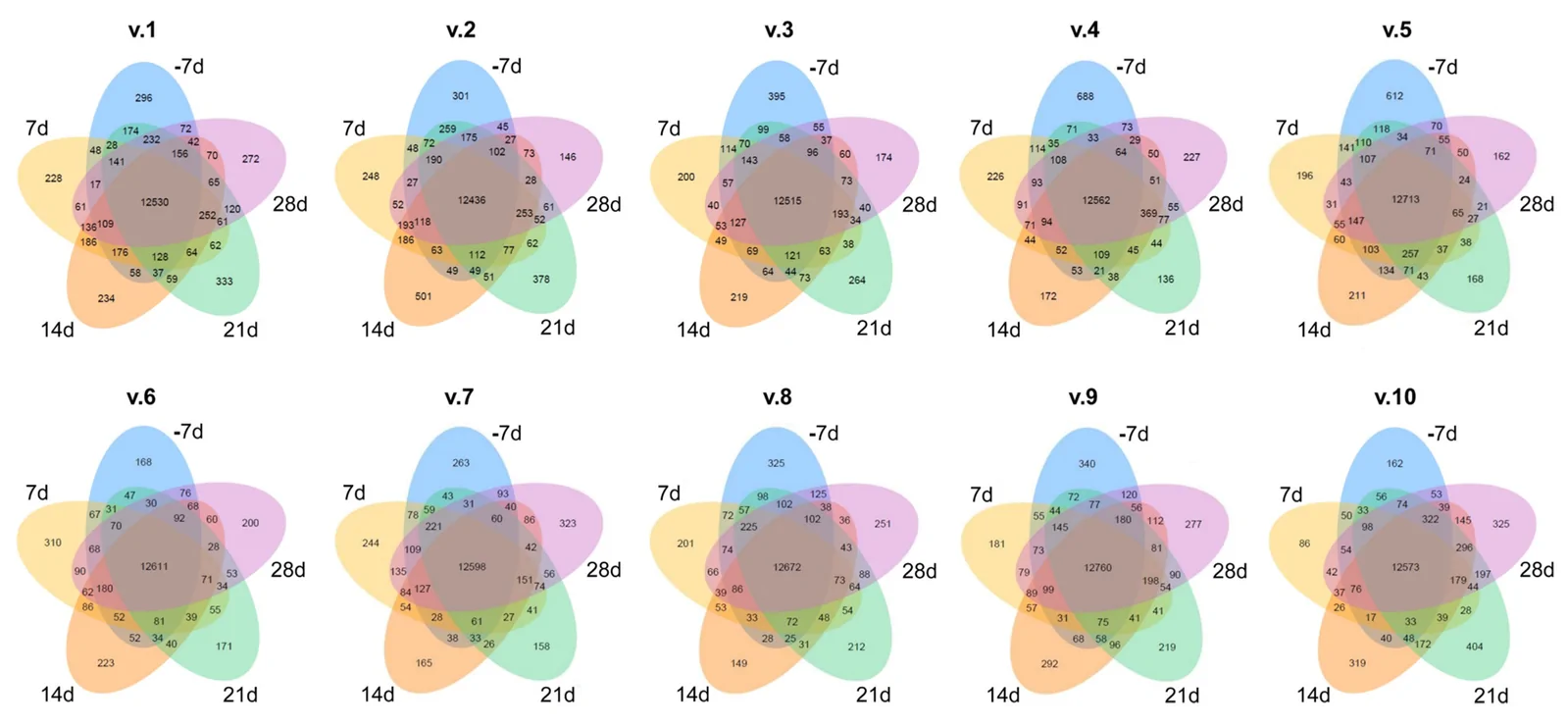
**Co-expression Venn diagrams of differentially expressed genes across five time points for each volunteer.** Petals: unique gene counts per time point. Overlaps: gene sets shared between ≥2 time points. The sum of the numbers within each ellipse represents the total number of DEGs detected at that time point.

Gene co-expression data were analyzed for each volunteer across all time points, and the results are shown as individual Venn diagrams for each of the 10 subjects (Figure 3). Each diagram’s petals quantify time-point-specific genes, with overlaps denoting shared genes across two or more time points.

### 3.5. Principal Component Analysis (PCA)

**Figure 4:**
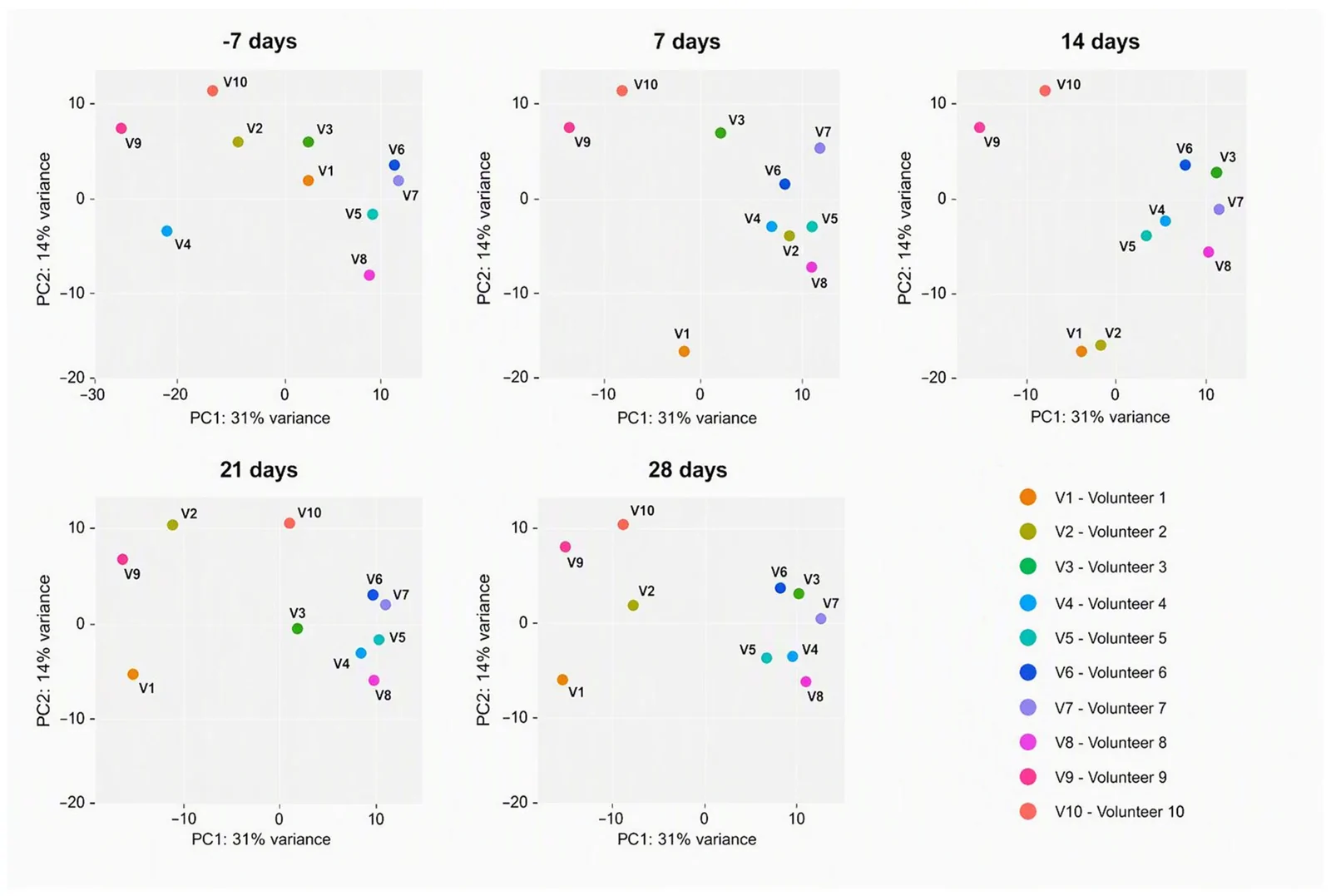
**Group principal component analysis (PCA) of transcriptomic profiles across the simulated microgravity (SMG) exposure time course.** Each point represents a single sample, and colors distinguish the ten volunteers. Samples cluster primarily according to volunteer identity rather than exposure stage, indicating that baseline transcriptional differences between individuals remain the dominant source of variance. Volunteers 3–8 consistently formed the main cluster, whereas volunteer 9 occupied a distinct region of PCA space throughout the study; volunteers 1, 2, and 10 were also distinct but positioned closer to the main cluster. Despite this pronounced stratification, a coordinated temporal response to SMG is still apparent: within each volunteer, samples from the exposure phase (days 7–21) are displaced relative to baseline (day −7), consistent with a common physiological adaptation, with the largest displacements at days 14 and 21, and some volunteers showing partial return toward baseline positions at day 28. The direction and extent of these displacements differ between volunteers, indicating substantial inter-individual heterogeneity in the response. PC1 and PC2 account for [X]% and [Y]% of the total variance, respectively.

PCA of the T cell transcriptomic data revealed that samples clustered primarily by volunteer identity rather than by exposure stage across all time points. Volunteers 3–8 consistently formed the main central cluster, whereas volunteer 9 occupied a distinct and highly separated region of the PCA space throughout the study; volunteers 1, 2, and 10 were also positioned outside the main cluster but remained comparatively closer to it. Despite this pronounced stratification by individual, coordinated shifts in PCA coordinates were observed between baseline (−7 days) and the immersion period (7–21 days) for all participants. The most pronounced displacements occurred at 14 and 21 days of exposure, while the 28-day samples partially reverted toward their respective pre-immersion positions (Figure 4).

## 4. Discussion

### 4.1. Cytokine–Cytokine Receptor Interaction

Ten DEGs (IL1B, IL7R, CXCR4, CXCL8, CSF1R, CSF2RB, CSF3R, TNF, TNFRSF1A, and TNFSF10) mapped to the KEGG cytokine–cytokine receptor interaction pathway (hsa04060) may indicate coordinated perturbation of cytokine ligand–receptor gene networks controlling inflammation, leukocyte trafficking, and hematopoiesis. MG consistently dysregulates cytokine networks and immune cell function, altering expression of pro-inflammatory IL-1β and TNF while shifting inflammatory set points that compromise host defense [13,14,15,16,17]. Recent single-cell data show invariant immune-suppression signatures under SMG or MG [18]. Genes encoding chemokine components CXCL8 and CXCR4 likely mediate MG-sensitive changes in lymphocyte chemotaxis and homing [14,16]. CSF1R, CSF2RB, CSF3R and IL7R potentially link to myeloid and lymphoid differentiation and hematopoietic output observed in spaceflight studies MG [14,17], whereas TNF, TNFRSF1A and TNFSF10 connect to apoptosis, survival, and inflammatory amplification [13,15,19], with NF-κB underscoring gravity sensitivity [20]. Importantly, no volunteer developed clinical signs of systemic inflammation during immersion, and body temperature remained within the normal range throughout (Methods 2.1; [10]). The mRNA-level changes reported here therefore arose in the absence of overt clinical symptoms, indicating that transcript-level responses to SMG can occur without detectable systemic inflammation.

### 4.2. T Cell Receptor Signaling

Eight DEGs (ICOS, JUN, FOS, MAP3K8, NFKBIA, NFKB2, TNF, and PIK3R1) mapping to the T cell receptor signaling pathway (hsa04660) may reflect dysregulation of gene networks involved in antigen–receptor proximal signaling, co-stimulation, transcriptional activation, and survival programs under MG. Perturbation of MAPK/NF-κB/AP-1 (JUN/FOS) nodes can blunt T cell activation and effector differentiation [18,21], with early studies demonstrating that MG inhibits immediate-early gene transcription via NF-κB signaling disruption [20,22]. PIK3R1 links mechanotransduction to T cell trafficking responses, altered activation thresholds and migratory behavior [19,23,24]. The sensitivity of these pathways to altered gravity was shown previously in primary human T cells under parabolic flight and clinorotation [25]. ICOS and MAP3K8 can further shape TCR signal amplitude, while changes in NF-κB components (NFKB2, NFKBIA) and TNF can shift the balance between proliferation, survival, and apoptosis under stress [21,22].

### 4.3. MAPK Signaling Pathway

Ten DEGs (JUND, JUN, FOS, MYC, GADD45A, GADD45B, DDIT3, MAP3K8, IL1B, and TNF) mapping to hsa04010 can point to a gravity-sensitive gene network coupling stress sensing, transcriptional reprogramming, and cell fate decisions. MAP3K8 and related upstream MAP3Ks determine ERK/JNK/p38 patterns, with combinatorial activation switching proliferation vs. apoptosis under MG [26]. Early work demonstrated that MAPK/SRC signaling is required for the mechano-induced expression of NF-κB [27], whereas AP-1 components (JUN/FOS/JUND) and MYC act as immediate-early effectors of MAPK signaling controlling cell cycle entry, metabolism, and survival under unloading and stress [28]. SMG was shown to disrupt the MAPK pathway in human BM-MSCs [29], and differentially regulated MAPK was observed in human T lymphocytes under MG [30]. Stress-responsive GADD45A/B and DDIT3 integrate DNA damage and ER stress with MAPK outputs, promoting growth arrest and apoptosis under SMG. IL1B and TNF link mechanical stress to cytokine amplification loops reshaping MAPK dynamics. Together, MG perturbs MAPK topology and timing via MAP3K, AP-1, MYC nodes and stress control, altering proliferation, survival, and inflammatory output [31].

### 4.4. Oncogenesis-Related Pathways

The following three cancer-associated KEGG pathways: Pathways in cancer (hsa05200), Transcriptional misregulation in cancer (hsa05202), and MicroRNAs in cancer (hsa05206)were among the top twelve most significant pathways identified in the BI analysis. A panel of DEGs was mapped to these pathways, revealing a coordinated alteration of gene networks involved in proliferation control, stress responses, survival vs. apoptosis decisions, cytoskeletal and extracellular matrix remodeling, inflammation, and post-transcriptional regulation.

hsa05200 contained 34 detected DEGs, of which 24 (70.6%) were hub genes, representing the highest enrichment among all analyzed pathways (Figure 2, Supplementary File S4). MG perturbs p53 gene networks and aging targets, linking altered gravity to DNA damage and stress signaling [7,32]. Previous studies have demonstrated that SMG significantly affects tumor growth patterns and drug responses in colorectal cancer organoid alterations [33]; NRAS/RAC2/PIK3R1 rewiring alters motility and cytoskeleton, spheroid formation and adhesion, and MYC-dependent drug/metabolic responses [34,35,36]; matrix remodeling genes (MMP9, FN1, and ITGA2B) provide bases for invasion, migration, immune interactions, and chemotherapy-response shifts [35,36,37,38]. Overall, the potential of using space-based investigations to inform cancer research on Earth has been increasingly recognized [37].

Twelve genes in hsa05202 (Supplementary File S3) are involved in EMT plasticity, inflammation, cell cycle, and survival. Recent syntheses of MG transcriptomics emphasize such transcriptional reprogramming as a hallmark of unloading [39]. SMG also produces a sustained defect in intestinal epithelial barrier function [40]. Innate immune and myeloid regulators together with inflammatory mediators link transcriptional misregulation to immune-stromal crosstalk in MG [39,41]. SMG inhibits osteogenesis via ZEB1 downregulation [42]. Unbalance of tumor suppressor and cell cycle checkpoints (TP53, CDKN1A, and CDKN1B) explains shifts in proliferation, 3D growth and stress response in MG models [43]. ZEB1 links EMT to replication-stress tolerance and DNA-damage vulnerabilities [41,44].

hsa05206 contained twelve genes (Supplementary File S3), highlighting miRNAs as post-transcriptional MG-related regulators. miR-34/miR-145 intersect MYC and TP53 gene networks; miR-21/miR-155 modulate MCL1 and SOCS1 nodes; and miR-200 regulates ZEB1 [45], suggesting that gravity-dependent miRNA changes can coordinate proliferation, apoptosis, EMT, and metabolism. Integrated miRNA and mRNA profiling of human peripheral blood lymphocytes revealed coordinated dysregulation of miRNA-mRNA networks under SMG [46]. MG studies report miRNA-network disruption associated with changes in CDKN1A/B, DDIT3, and MMP9, spheroid formation, drug sensitivity, stress tolerance [44,47]. In human lymphoblastoid cells, miRNAs expression changed significantly, with corresponding shifts in the transcription factors ETS1 and c-REL [48]. In primary human leukocytes, miR-21 induction during early T-cell activation was suppressed under MG, with coordinate down-regulation of its target genes [49]. 

Collectively, these pathways may show conserved oncogenesis-related reprogramming at transcriptional, post-transcriptional, and gene network levels, converging on TP53, MYC, NF-κB, AP-1, and PI3K and affecting homeostasis, regeneration, and cancer phenotypes in space biology [7,32,50].

### 4.5. Apoptosis Pathway

Seventeen DEGs were mapped to the Apoptosis pathway (hsa04210) (Supplementary File S3) involved in multilayered control of survival vs. death via mitochondrial, death receptor, ER stress, and pro-survival kinase axes. Mitochondrial apoptosis (CYCS, MCL1, and BCL2A1) is redox and mechanical stress sensitive and repeatedly implicated in MG-enhanced apoptosis [51,52,53,54,55]. Altered gravity affects stress-responsive effectors (TP53, GADD45 family, CDKN1A, and DDIT3) that integrate DNA damage and ER stress to promote repair or mitochondrial death pathways [52,56,57,58]. TNF, TNFRSF1A, TNFSF10, and NFKBIA provide inputs that reinforce apoptosis or engage survival. PIK3R1/CSF2RB link cytokine trophic signaling to AKT/ERK survival, and reported caspase activation and ER stress markers are consistent with this gene network model [51,52,53,54].

### 4.6. Cell Cycle Regulation Pathway

Seven cell cycle DEGs (CDKN1A/B, GADD45A/B/G, MYC, and TP53) in hsa04110 may point to coordinated disruption of G1/S checkpoint control, DNA-damage sensing, and replication stress responses. CDKN1A is repeatedly elevated in mechanically sensitive tissues during spaceflight and SMG, correlating with arrest, reduced proliferation, and impaired regeneration. CDKN1A modulation alters outcomes in rodent flight models [7,53,59,60,61,62]. GADD45 couple DNA damage and environmental stress to checkpoints and p38/MAPK, enforcing arrest or repair in MG [59,63]. TP53-MYC interplay determines transient arrest, senescence, or apoptosis where MG can perturb p53 and MYC signaling [7,34]. CDKN1A, GADD45s, TP53, and MYC are considered as candidate countermeasure targets for preserving tissue regenerative potential during long-duration missions.

### 4.7. FoxO Signaling Pathway

IL7R, IRS2, and TNFSF10 in the MG signature mapping to FoxO signaling (hsa04068) may indicate a conserved stress axis reprogramming survival, metabolism, and immune homeostasis. FoxO integrates growth factor and insulin-PI3K-AKT inputs whereas IRS2 links insulin and IGF signaling to AKT and FoxO phosphorylation, so MG-related IRS2 changes could shift FoxO control of oxidative defenses, autophagy, and metabolism [64]. IL7R intersects FoxO-regulated lymphoid survival and homeostatic proliferation, so altered IL7R in MG may perturb FoxO-dependent maintenance of T and B cell pools [65,66]. TNFSF10 engages extrinsic apoptosis modulated by FoxO targets, tuning adaptive stress vs. death in MG [67] as well as in acute and chronic neurological disorders [68]. Thus, above connections suggest that MG-induced shifts IRS2, IL7R, and TNFSF10 signaling converge on FoxO transcriptional control of redox homeostasis, cell cycle arrest, autophagy, and apoptosis [64,66,69].

### 4.8. Primary Immunodeficiency

ICOS, IL7R, and BTK in Primary immunodeficiency (hsa05340) suggest that MG may affect lymphocyte development, co-stimulation, and BCR signaling, potentially phenocopying primary immunodeficiency. IL7R is essential for lymphoid progenitor survival and differentiation, and its activity drives human B cell progenitor expansion and V(D)J usage, so altered IL7R may reduce lymphopoiesis and repertoire diversity [65]. BTK transduces BCR signals for maturation and antibody responses, so its dysregulation impairs humoral immunity [70]. ICOS supports Tfh function and GC responses, so dysregulation may compromise B-T collaboration and long-lived antibody production [71]. Spaceflight causes persistent adaptive immune alterations, impaired vaccine responses, and altered T cell differentiation [72,73], as well as T cell activation and differentiation sensitive to MG [74]. These genes link transcriptome changes to reduced adaptive immune competence in spaceflight and SMG [14,65,70].

### 4.9. Osteoclast Differentiation

Fourteen DEGs mapped to the Osteoclast differentiation (hsa04380) (Supplementary File S3) may indicate a module connecting inflammatory cues, receptor kinase inputs, transcriptional effectors, and NF-κB/PI3K programs to the generation and activation of bone-resorbing osteoclasts. CSF1R signaling and pro-inflammatory TNF and IL1B drive osteoclast lineage commitment and fusion, and their frequent dysregulation in MG explains the enhanced osteoclastogenesis and accelerated bone resorption observed in unloaded models and astronauts [75,76,77]. SMG increases the RANKL/OPG ratio to promote osteoclastogenesis [78], with downstream MAPK AP-1 components (JUN, FOS, and JUND) and NF-κB regulators (NFKBIA, NFKB2) cooperating with SPI1 to induce osteoclast-specific programs, while PIK3R1 and BTK integrate trophic and receptor signals to modulate survival, cytoskeletal reorganization, and resorptive activity [70,79].Systems analyses show coordinated osteoclast and osteoblast responses to SMG [80], and perturbation of negative feedback modulator SOCS1 and STAT signaling in MG, which tune differentiation, can shift the balance toward excessive resorption [19,77].Thus, MG-induced cytokine imbalance, altered CSF1R/BTK/PI3K signaling, and reprogrammed AP-1/NF-κB transcriptional responses converge to promote osteoclast differentiation and resorptive activity, consistent with documented MG-associated bone loss and osteoclastogenic pathways [75,76,77,79,80].

### 4.10. Regulation of Actin Cytoskeleton

Actin-cytoskeleton regulation (hsa04810) enrichment (Supplementary File S3) may link altered gravitational loading to adhesion, force transmission and motility reorganization. Integrin-ECM complexes (ITGA2B/ITGAM; FN1) anchor actin networks and focal adhesions, and their unloading-disrupted reciprocity remodels focal adhesions and RAS/PI3K/Rho GTPase signaling, altering lamellipodial dynamics, phagocytic cup formation, and cell spreading in immune and stromal cells under SMG [81,82,83,84,85,86]. RAC2/NRAS coordinate actin nucleation and membrane protrusion, and RAC1/RAC2 are essential for immune motility and antigen responses, linking RAC2 to impaired chemotaxis and altered immune function [12,86]. PIK3R1 couples integrin and growth factor receptors to WASP/WAVE/ARP2/3 and metabolic programs sustaining actin turnover [23,83,84], while nuclear WASP modulates T cell transcriptional programs, connecting actin regulators to lineage-specific output [87]. Rho GTPases are key mechanotransducers in MG, linking cytoskeletal dynamics to downstream signaling [88], and SMG responses converge on cytoskeletal and adhesion modules distinct from those induced by hydrodynamic stress [12,89]. T cell dynamics confirm the centrality of cytoskeletal regulation in gravity sensing [7,90], supporting a mechanotransductive axis in which integrin-ECM disruption, RAS/PI3K-Rac signaling, and actin-nuclear coupling translate MG into transcriptional reprogramming [86,89,91].

### 4.11. PCA-Based Inter-Individual Stratification and Heterogeneous SMG Response Trajectories

The PCA trajectories suggest a strong inter-individual effect that is substantially larger than the group-level effect of SMG. At all time points, samples cluster primarily according to volunteer identity rather than according to exposure stage, indicating that baseline transcriptional differences between individuals remain a dominant source of variance. Specifically, volunteers 3–8 consistently formed the main cluster, whereas volunteer 9remains highly separated and occupies a distinct region of PCA space throughout the study. Volunteers 1, 2 and 10 are also distinct but somewhat closer to the main cluster. Despite this pronounced stratification, a temporal response to SMG is still apparent. Between baseline (−7 days) and exposure (7–21 days), all individuals display coordinated shifts in PCA space, suggesting a common physiological adaptation. The movement is most pronounced during immersion (particularly at 14 and 21 days), whereas at 28 days the samples partially return toward their initial positions, consistent with recovery following cessation of the intervention. However, the magnitude and direction of these trajectories differ among volunteers, indicating substantial heterogeneity in response. Overall, the PCA supports the conclusion that SMG induces a detectable transcriptomic effect, but that the response is strongly modulated by individual-specific factors, leading to marked stratification of the cohort and variable adaptation dynamics between volunteers.

These findings are consistent with human bed-rest transcriptomic studies, which demonstrate that while a core set of genes responds consistently to unloading, baseline expression profiles are highly individual-specific and remain the primary determinant of variance throughout the intervention [92]. This observation aligns with the broader framework proposed for space medicine, wherein omics-based analyses are essential to capture individual molecular profiles and develop tailored countermeasures [93]. Furthermore, the marked heterogeneity observed in our cohort highlights the growing understanding that genetic variability significantly modulates physiological responses to microgravity, supporting the need for a shift from universal to personalized strategies for astronaut health management during long-duration missions [92,93,94].

### 4.12. Study Limitations and Future Directions

This study has several limitations.

First, the cohort of volunteers was small (n = 10) and composed exclusively of healthy males aged 24–32 years. This may have contributed to the inter-individual heterogeneity observed, and it limits the extent to which our findings can be generalized to female, younger, older, or clinically compromised individuals.

Second, not all genes returned to baseline by day 28, suggesting that a longer recovery period may be needed to resolve the transcriptional changes induced during simulated microgravity. Future studies would benefit from an extended follow-up period beyond day 28, for example, an additional time point four weeks after the end of the immersion period, to evaluate the complete return of RNA expression to baseline levels.

Third, the present analysis did not resolve CD4 and CD8 T cell subsets separately. Future studies in larger cohorts, combining functional immune assays with single-cell transcriptomics, will be required to resolve subset-specific responses and to determine whether the transcriptional changes observed here correspond to functional changes in T cell activation and effector function, which will be important for designing interventions to mitigate adverse transcriptional trends.

Fourth, functional immune assays, hormone quantification, and protein-level measurements were not performed. The absence of functional readouts and endocrine data limits our ability to link the observed transcriptional changes to functional immune outcomes or to the hormonal changes associated with dry immersion, and the absence of proteomic or phosphoproteomic data means that pathway activity cannot be inferred from transcriptomic data alone. Incorporating these aspects, and relating the observed molecular changes to physiological and clinical outcomes in the same individuals, will be an important objective for future studies.

Fifth, although CD3+ T cells were isolated with approximately 95% purity using an identical protocol for all samples and time points, residual non-T cell contamination cannot be fully excluded and may have contributed to the observed transcriptional signal, particularly for genes expressed at high levels in non-T cell populations. Future studies would benefit from per-sample purity verification and, where feasible, single-cell approaches that allow cell-type assignment.

Sixth and finally, the potential clinical application of these findings remains speculative. While transcriptomic profiling may eventually contribute to personalized countermeasures for astronauts, RNA expression signatures alone are not currently sufficient to guide prophylactic prescribing, and substantially more data, including functional validation in larger cohorts, will be required.

## 5. Conclusions

### 5.1. T Cell Transcriptomes Demonstrate Dynamic Pathway-Specific Responses to Microgravity

Gene expression profiles and temporal trajectories confirm that T cell transcriptional profiles undergo a dynamic pathway-specific, time-dependent adaptation to microgravity, rather than a static or monotonic shift. Coordinated displacements from background (−7 days) through the exposure period (1–21 days) indicate a shared transcriptional response that peaks during the middle-to-late exposure phase. The subsequent partial return toward baseline positions by day 28 further supports a recovery trajectory, underscoring that the transcriptomic impact of microgravity is partially reversible.

### 5.2. T Cell Transcriptome Modulation by Microgravity In Vivo Is Associated with Key Immune and Oncogenic Gene Networks

In addition to global expression shifts, the PCA-defined variance points to biologically meaningful transcript-level changes in genes mapping to specific regulatory pathways. The involvement of oncogenic and immunoregulatory networks is particularly noteworthy, as these gene landscapes are critical for maintaining T cell homeostasis and functional integrity. This suggests that microgravity does not simply induce general transcriptional noise but rather has targeted effects on transcripts within molecular pathways linking immunity, cell growth control, and stress-response, with potential implications for host defense and long-term cellular health.

### 5.3. T Cell Transcriptome Pathway-Specific Responses to Microgravity In Vivo Are Individually Variable

Despite the presence of a common temporal signature, the magnitude and direction of these pathway-specific responses are strongly modulated by baseline inter-individual differences. The stratification of the cohort demonstrates that volunteer identity is the dominant source of transcriptional variance. This marked heterogeneity implies that the same microgravity stimulus elicits divergent gene expression outcomes across individuals, likely driven by pre-existing baseline transcriptomic profiles. Therefore, studies of T cell adaptation to spaceflight must account for significant interindividual variability, highlighting the need for personalized biomarkers and tailored countermeasures in future space missions.

## Figures and Tables

**Table 1:** Top 12 KEGG pathways with the highest statistical significance.

#	KEGG Pathway	Pathway ID
1	Cytokine–cytokine receptor interaction	hsa04060
2	T cell receptor signaling	hsa04660
3	MAPK signaling pathway	hsa04010
4	Pathways in cancer	hsa05200
5	Transcriptional misregulation in cancer	hsa05202
6	MicroRNAs in cancer pathways	hsa05206
7	Apoptosis	hsa04210
8	Cell cycle regulation	hsa04110
9	FoxO signaling	hsa04068
10	Primary immunodeficiency	hsa05340
11	Osteoclast differentiation	hsa04380
12	Regulation of actin cytoskeleton	hsa04810

**Table 2:** Forty-four differentially expressed hub genes found in top 12 KEGG pathways.

Ensemble ID	Gene	Gene Name	Gene ID
ENSG00000125538	IL1B	interleukin 1 beta	3553
ENSG00000168685	IL7R	interleukin 7 receptor	3575
ENSG00000121966	CXCR4	C-X-C motif chemokine receptor 4	7852
ENSG00000169429	CXCL8	C-X-C motif chemokine ligand 8	3576
ENSG00000182578	CSF1R	colony stimulating factor 1 receptor	1436
ENSG00000100368	CSF2RB	colony stimulating factor 2 receptor subunit beta	1439
ENSG00000119535	CSF3R	colony stimulating factor 3 receptor	1441
ENSG00000232810	TNF	tumor necrosis factor	7124
ENSG00000067182	TNFRSF1A	TNF receptor superfamily member 1A	7132
ENSG00000121858	TNFSF10	TNF superfamily member 10	8743
ENSG00000163600	ICOS	inducible T cell costimulator	29851
ENSG00000177606	JUN	Jun proto-oncogene, AP-1 transcription factor subunit	3725
ENSG00000170345	FOS	Fos proto-oncogene, AP-1 transcription factor subunit	2353
ENSG00000107968	MAP3K8	mitogen-activated protein kinase kinase kinase 8	1326
ENSG00000100906	NFKBIA	NFKB inhibitor alpha	4792
ENSG00000077150	NFKB2	nuclear factor kappa B subunit 2	4791
ENSG00000145675	PIK3R1	phosphoinositide-3-kinase regulatory subunit 1	529
ENSG00000010671	BTK	Bruton tyrosine kinase	695
ENSG00000130522	JUND	JunD proto-oncogene, AP-1 transcription factor subunit	3727
ENSG00000136997	MYC	MYC proto-oncogene, bHLH transcription factor	4609
ENSG00000116717	GADD45A	growth arrest and DNA damage-inducible alpha	1647
ENSG00000099860	GADD45B	growth arrest and DNA damage-inducible beta	4616
ENSG00000130222	GADD45G	growth arrest and DNA damage-inducible gamma	10912
ENSG00000175197	DDIT3	DNA damage-inducible transcript 3	1649
ENSG00000066336	SPI1	Spi-1 proto-oncogene	6688
ENSG00000213281	NRAS	NRAS proto-oncogene, GTPase	4893
ENSG00000128340	RAC2	Rac family small GTPase 2	5880
ENSG00000115415	STAT1	signal transducer and activator of transcription 1	6772
ENSG00000141510	TP53	tumor protein p53	7157
ENSG00000124762	CDKN1A	cyclin-dependent kinase inhibitor 1A	1026
ENSG00000111276	CDKN1B	cyclin-dependent kinase inhibitor 1B	1027
ENSG00000100985	MMP9	matrix metallopeptidase 9	4318
ENSG00000115414	FN1	fibronectin 1	2335
ENSG00000005961	ITGA2B	integrin subunit alpha 2b	3674
ENSG00000073756	PTGS2	prostaglandin-endoperoxide synthase 2	5743
ENSG00000186350	RXRA	retinoid X receptor alpha	6256
ENSG00000172115	CYCS	cytochrome c, somatic	54205
ENSG00000169896	ITGAM	integrin subunit alpha M	3684
ENSG00000148516	ZEB1	zinc finger E-box binding homeobox 1	6935
ENSG00000170458	CD14	CD14 molecule	929
ENSG00000140379	BCL2A1	BCL2-related protein A1	597
ENSG00000143384	MCL1	MCL1 apoptosis regulator, BCL2 family member	4170
ENSG00000185338	SOCS1	suppressor of cytokine signaling 1	8651
ENSG00000185950	IRS2	insulin receptor substrate 2	866

## Data Availability

The data that support the findings of this study are currently available in the NCBI GEO public repository under accession number GSE301964: https://www.ncbi.nlm.nih.gov/geo/ (accessed on 1 October 2026) [Gene Expression Omnibus] [https://www.ncbi.nlm.nih.gov/geo/query/acc.cgi?acc=GSE301964 (accessed on 1 October 2026)] [GSE301964].

## Supplementary Materials

The following supporting information can be downloaded at https://www.mdpi.com/article/10.3390/cells15212017/s1, Supplementary File S1. Normalized expression of differentially expressed genes per volunteer and time point. Supplementary File S2. Top12 KEGG pathways. Supplementary File S3. 44 hub genes differentially expressed in at least two of top 12 KEGG pathways. Supplementary File S4. KEGG pathways in cancer (hsa05200) DEGs. Supplementary File S5. iThenticate report_31072026. Supplementary File S6. Expression of 10 hub genes distributed across nine of top 12 KEGG pathways. Supplementary File S7. RNA-seq data QC. Supplementary File S8. List of software used for RNA-seq data analysis.

## Abbreviations

BG	background
MG	microgravity
PBMC	peripheral blood mononuclear cells
PCA	principal component analysis
QC	quality control
SMG	simulated microgravity
