# Supplementary material for: T Cell Transcriptomes Are Dynamically Modulated by Microgravity In Vivo Along Core Gene Networks and Key Molecular Pathways
Source: Cells. 2026 Nov 5;15(21):2017. doi: 10.3390/cells15212017 (PMC13761565; doi:10.3390/cells15212017)
Supplement: Supplementary file 1 [file cells-15-02017-s001.zip › cells-4505839-supplementary.pdf]

Supplementary File S1

Normalized expression of differentially expressed genes per volunteer & timepoint

Normalized expression of differentially expressed genes: volunteer 1

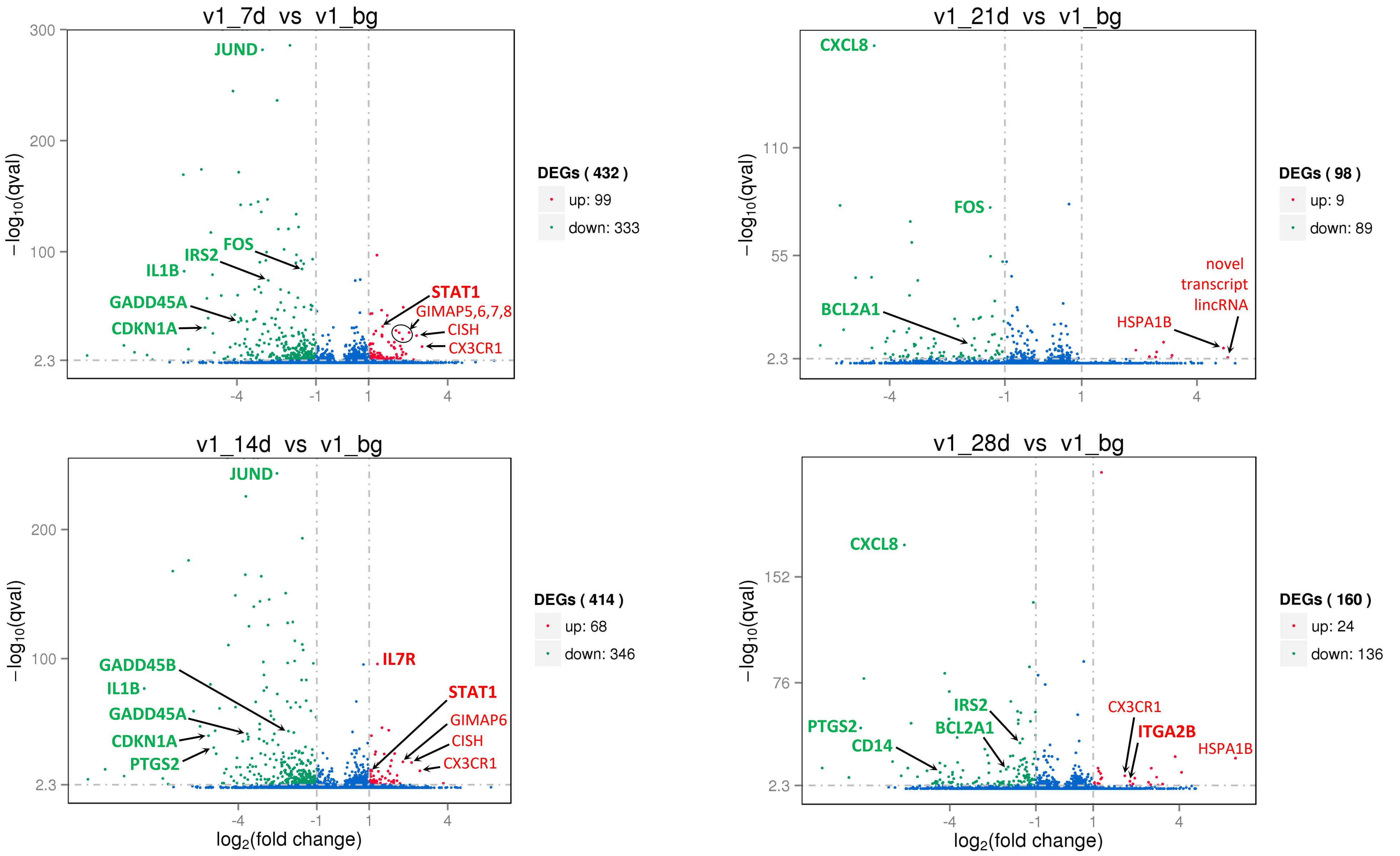

Normalized expression of differentially expressed genes: volunteer 2

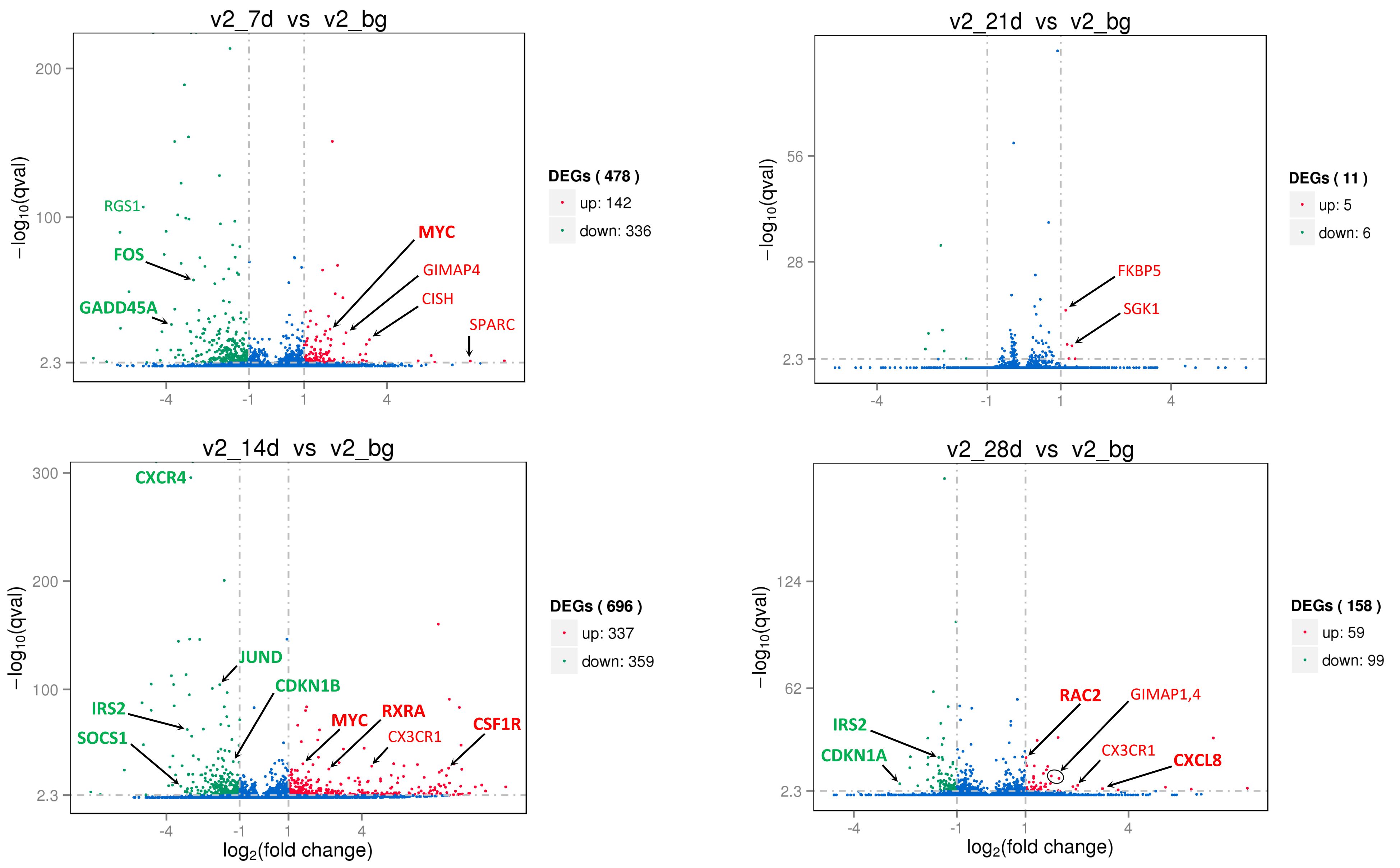

Normalized expression of differentially expressed genes: volunteer 3

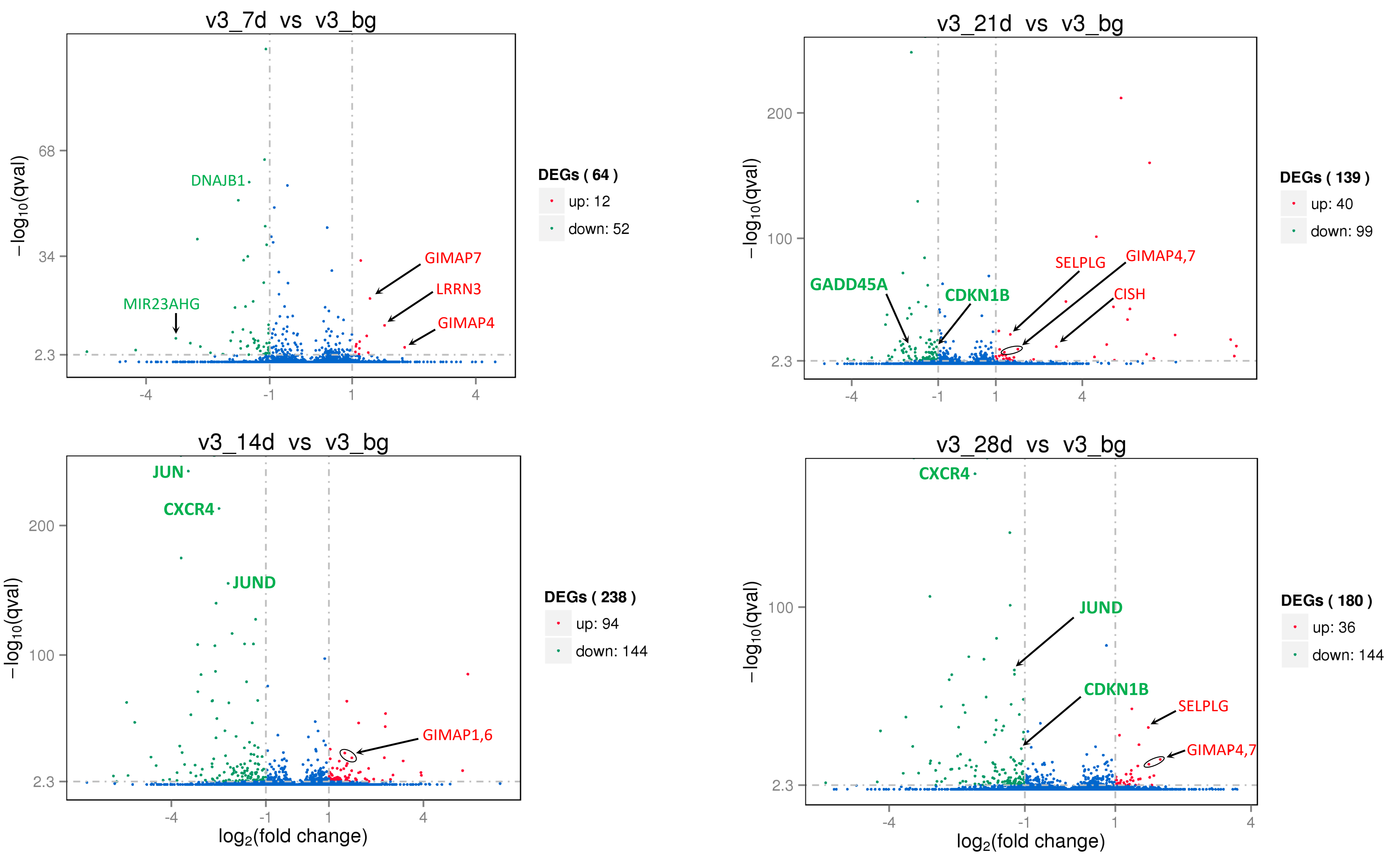

Normalized expression of differentially expressed genes: volunteer 4

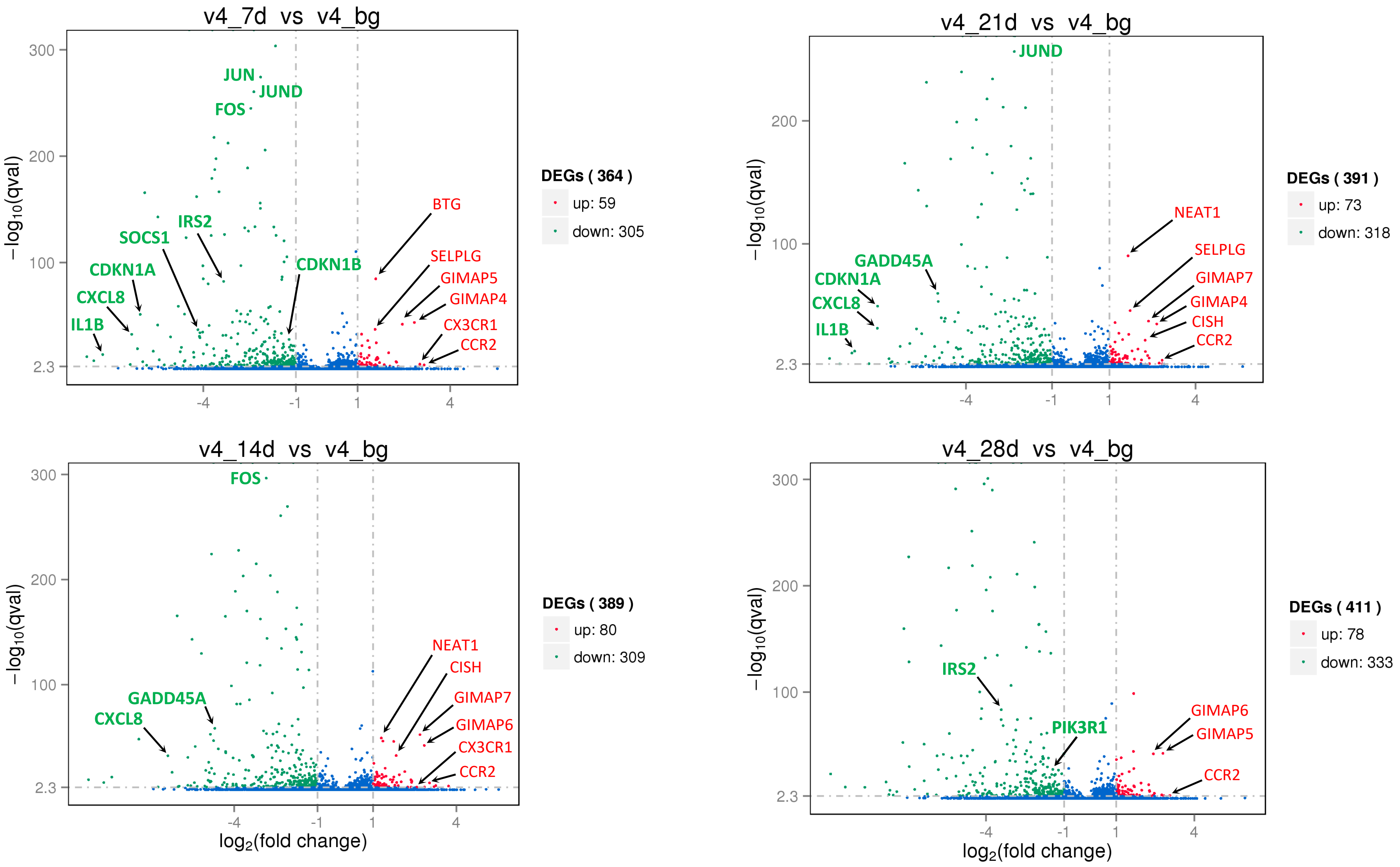

Normalized expression of differentially expressed genes: volunteer 5

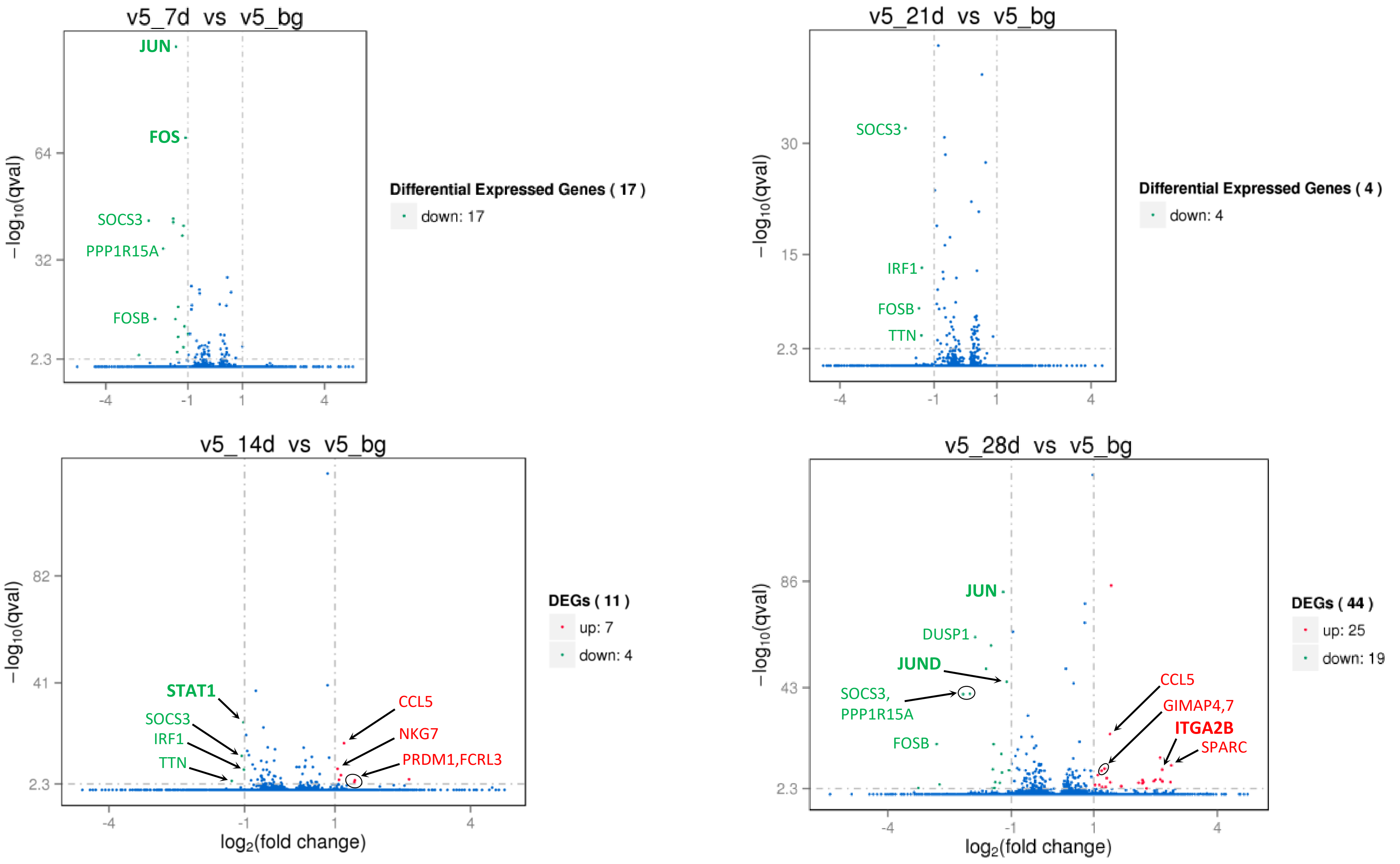

Normalized expression of differentially expressed genes: volunteer 6

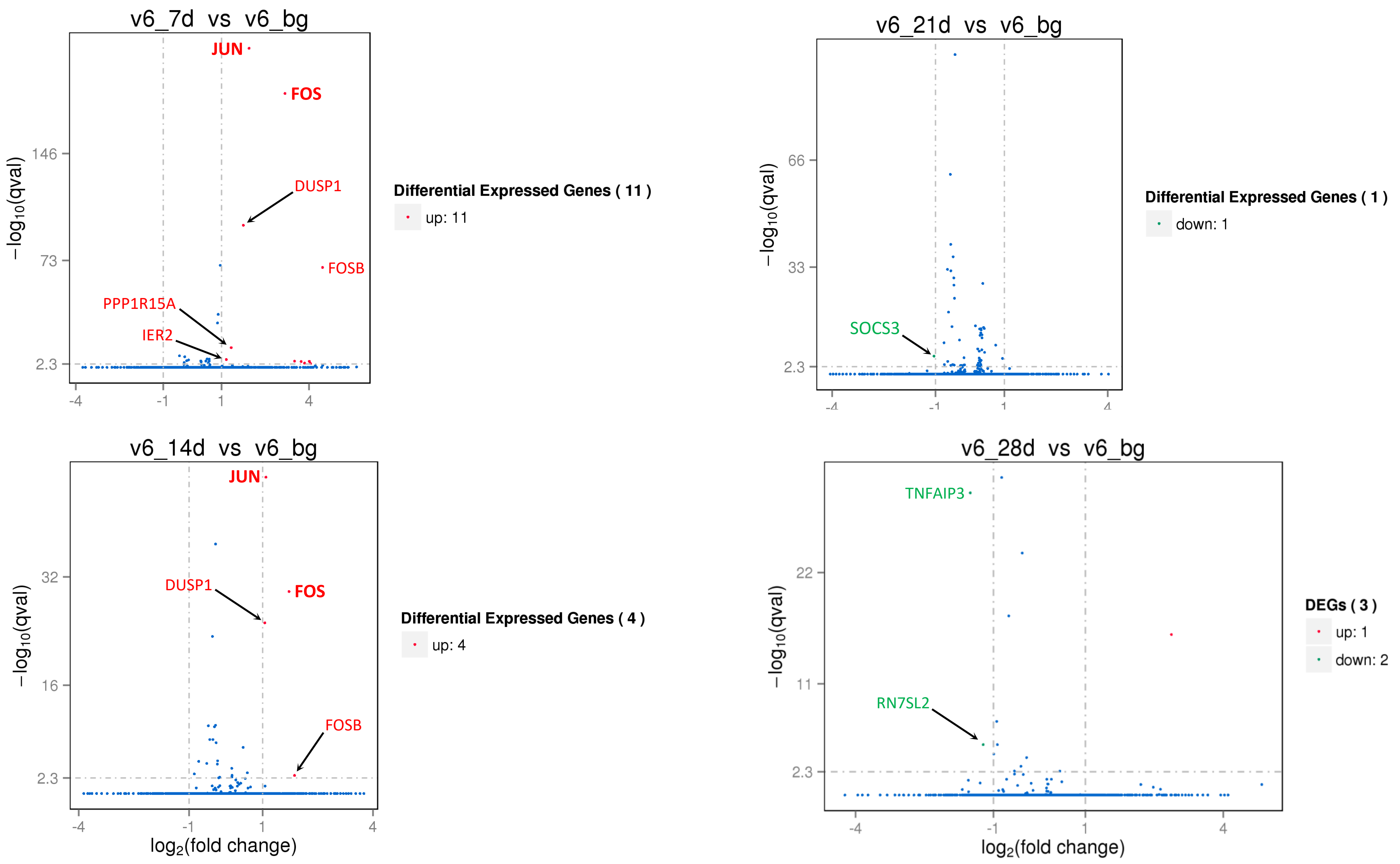

Normalized expression of differentially expressed genes: volunteer 7

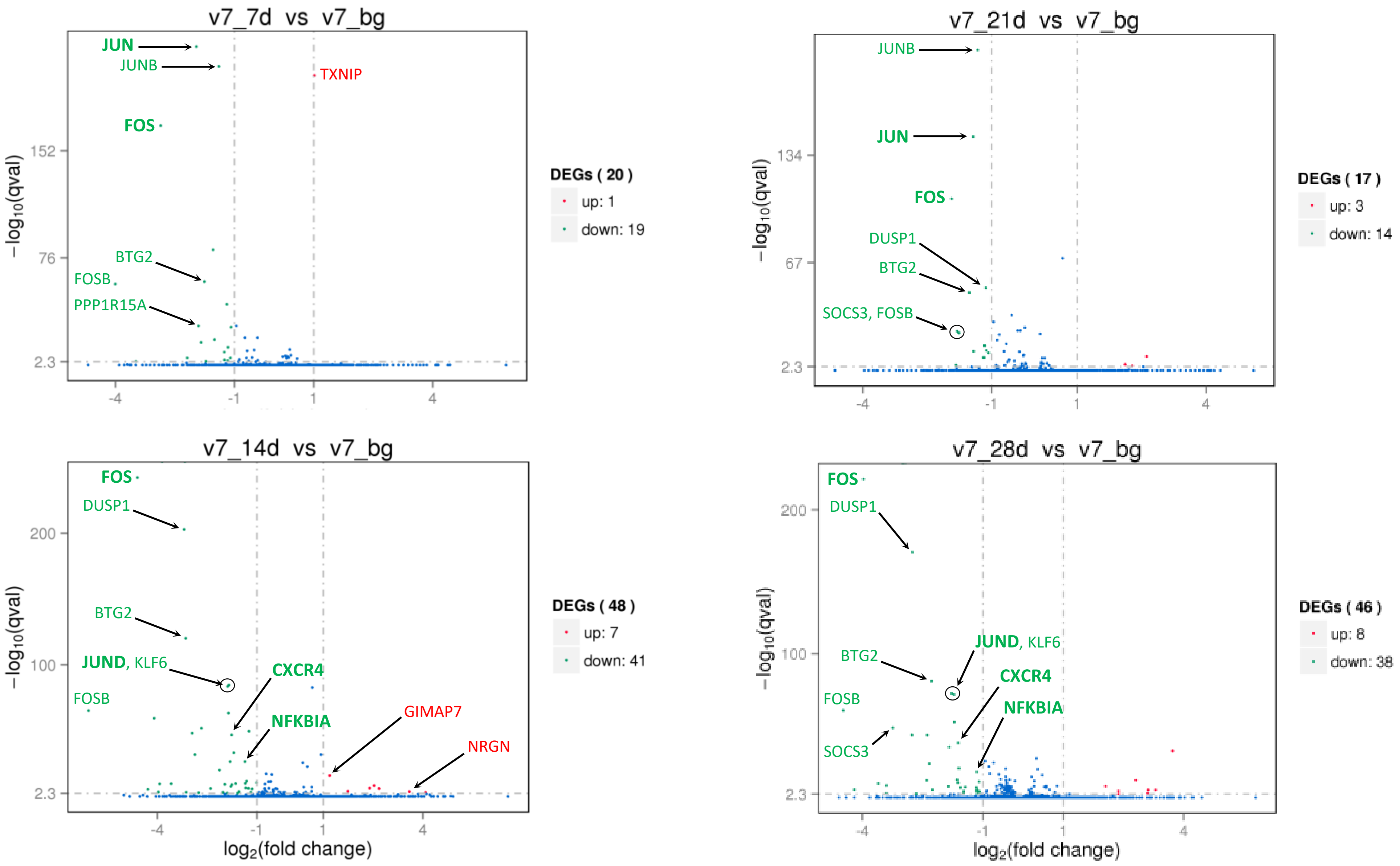

Normalized expression of differentially expressed genes: volunteer 8

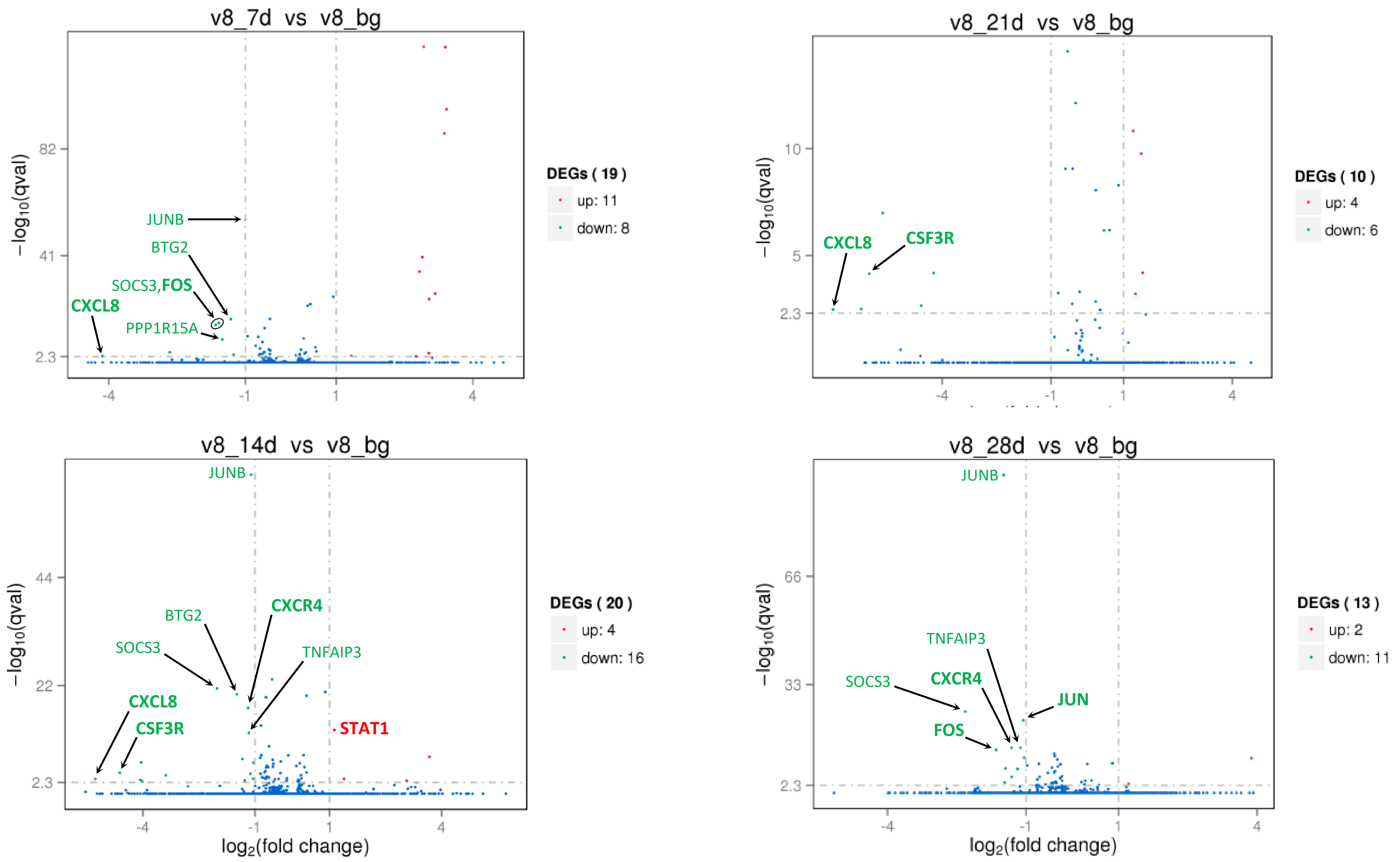

Normalized expression of differentially expressed genes: volunteer 9

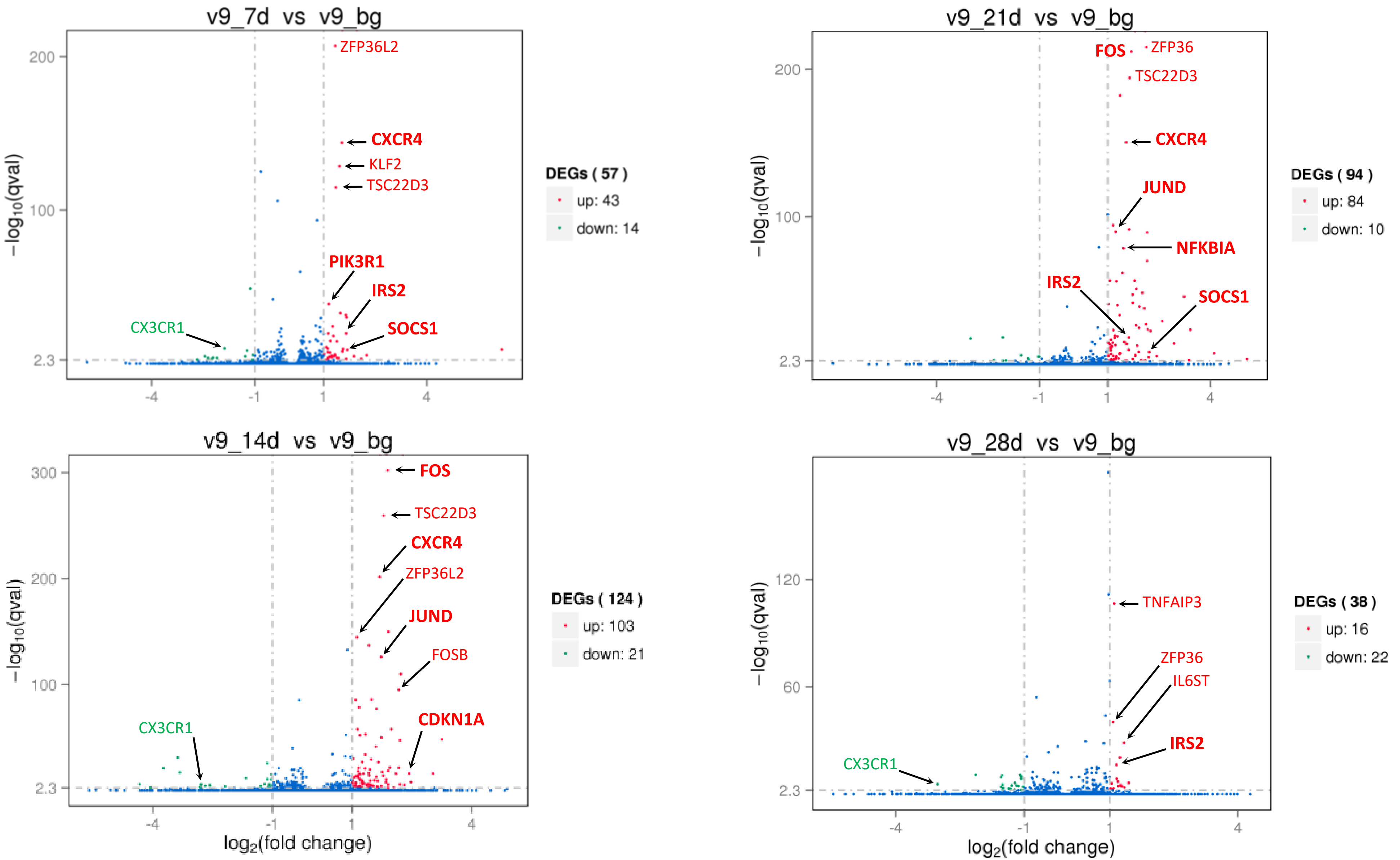

Normalized expression of differentially expressed genes: volunteer 10

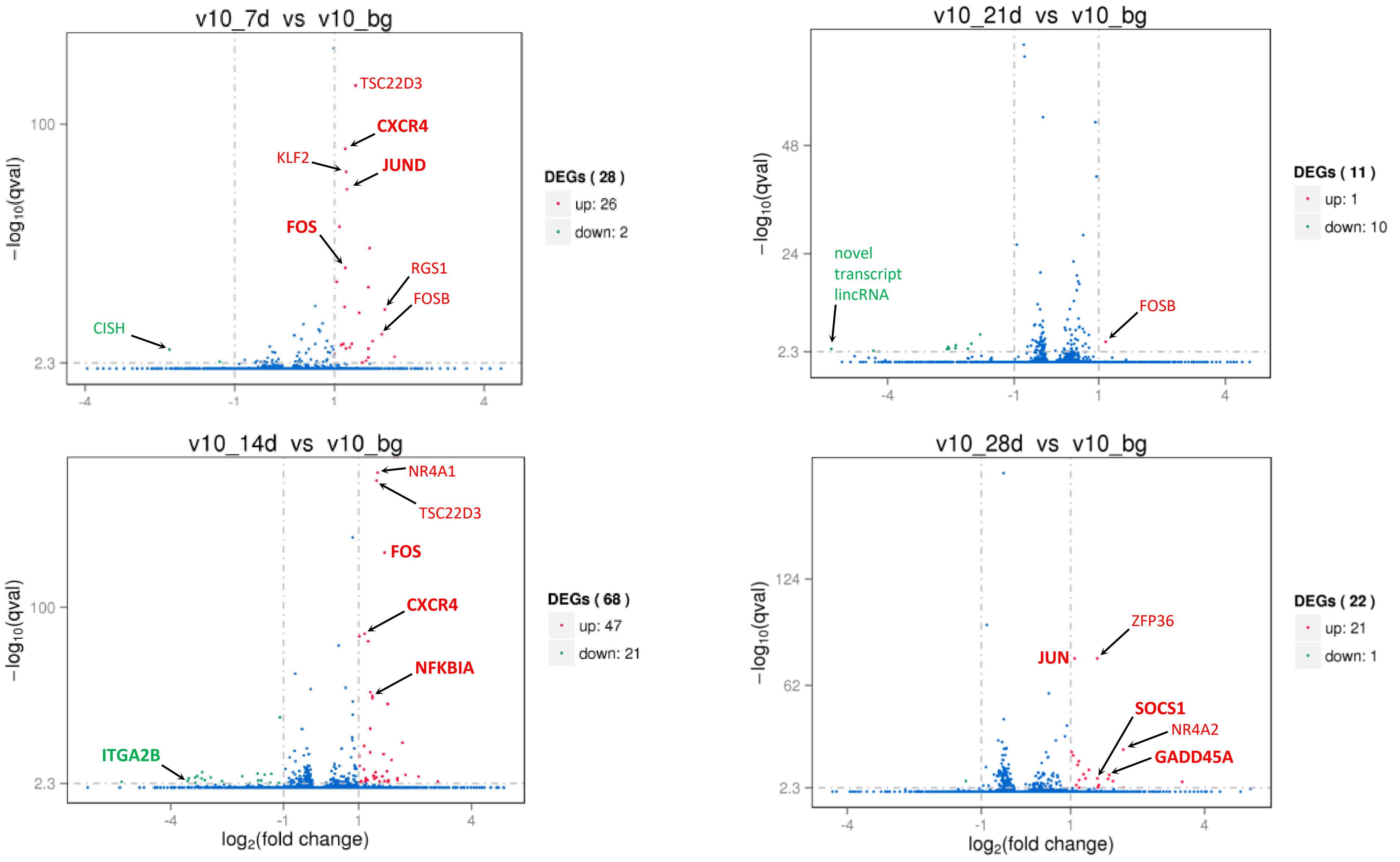

Supplementary File S2 **KEGG pathway enrichment**

#	Term	ID	Corrected P-Value	Database	Hyperlink
1	Cytokine-cytokine receptor interaction	hsa04060	2.789E-06	KEGG PATHWAY	https://www.kegg.jp/kegg-bin/show_pathway?hsa04060
2	T cell receptor signaling	hsa04660	4.466E-07	KEGG PATHWAY	https://www.kegg.jp/kegg-bin/show_pathway?hsa04660
3	MAPK signaling pathway	hsa04010	9.597E-10	KEGG PATHWAY	https://www.kegg.jp/kegg-bin/show_pathway?hsa04010
4	Pathways in cancer	hsa05200	5.454E-08	KEGG PATHWAY	https://www.kegg.jp/kegg-bin/show_pathway?hsa05200
5	Transcriptional misregulation in cancer	hsa05202	4.100E-08	KEGG PATHWAY	https://www.kegg.jp/kegg-bin/show_pathway?hsa05202
6	MicroRNAs in cancer pathways	hsa05206	4.779E-04	KEGG PATHWAY	https://www.kegg.jp/kegg-bin/show_pathway?hsa05206
7	Apoptosis	hsa04210	1.031E-11	KEGG PATHWAY	https://www.kegg.jp/kegg-bin/show_pathway?hsa04210
8	Cell cycle regulation	hsa04110	1.947E-03	KEGG PATHWAY	https://www.kegg.jp/kegg-bin/show_pathway?hsa04110
9	FoxO signaling	hsa04068	1.406E-09	KEGG PATHWAY	https://www.kegg.jp/kegg-bin/show_pathway?hsa04068
10	Primary immunodeficiency	hsa05340	1.919E-04	KEGG PATHWAY	https://www.kegg.jp/kegg-bin/show_pathway?hsa05340
11	Osteoclast differentiation	hsa04380	1.406E-09	KEGG PATHWAY	https://www.kegg.jp/kegg-bin/show_pathway?hsa04380
12	Regulation of actin cytoskeleton	hsa04810	1.804E-03	KEGG PATHWAY	https://www.kegg.jp/kegg-bin/show_pathway?hsa04810

Supplementary File S3

44 hub genes differentially expressed in at least two of top 12 KEGG pathways

Ensemble ID	Gene	Gene Name	Gene ID	Cytokine-cytokine receptor interaction	T cell receptor signaling	MAPK signaling pathway	Pathways in cancer	Transcriptional misregulation in cancer	MicroRNAs in cancer pathways	Apoptosis	Cell cycle regulation	FoxO signaling	Primary immunodeficiency	Osteoclast differentiation	Regulation of actin cytoskeleton
ENSG00000125538	IL1B	interleukin 1 beta	3553	■		■								■	
ENSG00000168685	IL7R	interleukin 7 receptor	3575	■								■	■		
ENSG00000121966	CXCR4	C-X-C motif chemokine receptor 4	7852	■			■								
ENSG00000169429	CXCL8	C-X-C motif chemokine ligand 8	3576	■			■								
ENSG00000182578	CSF1R	colony stimulating factor 1 receptor	1436	■			■	■						■	
ENSG00000100368	CSF2RB	colony stimulating factor 2 receptor subunit beta	1439	■						■					
ENSG00000119535	CSF3R	colony stimulating factor 3 receptor	1441	■			■								
ENSG00000232810	TNF	tumor necrosis factor	7124	■	■	■				■				■	
ENSG00000067182	TNFRSF1A	TNF receptor superfamily member 1A	7132	■						■				■	
ENSG00000121858	TNFSF10	TNF superfamily member 10	8743	■						■		■			
ENSG00000163600	ICOS	inducible T cell costimulator	29851		■								■		
ENSG00000177606	JUN	Jun proto-oncogene, AP-1 transcription factor subunit	3725		■	■	■			■				■	
ENSG00000170345	FOS	Fos proto-oncogene, AP-1 transcription factor subunit	2353		■	■	■			■				■	
ENSG00000107968	MAP3K8	mitogen-activated protein kinase kinase kinase 8	1326		■	■									
ENSG00000100906	NFKBIA	NFKB inhibitor alpha	4792		■		■			■				■	
ENSG00000077150	NFKB2	nuclear factor kappa B subunit 2	4791		■		■							■	
ENSG00000145675	PIK3R1	phosphoinositide-3-kinase regulatory subunit 1	529		■					■				■	■
ENSG00000010671	BTK	Bruton tyrosine kinase	695										■	■	
ENSG00000130522	JUND	JunD proto-oncogene, AP-1 transcription factor subunit	3727			■								■	
ENSG00000136997	MYC	MYC proto-oncogene, bHLH transcription factor	4609			■	■	■	■		■				
ENSG00000116717	GADD45A	growth arrest and DNA damage inducible alpha	1647			■				■	■				
ENSG00000099860	GADD45B	growth arrest and DNA damage inducible beta	4616			■				■	■				
ENSG00000130222	GADD45G	growth arrest and DNA damage inducible gamma	10912							■	■				
ENSG00000175197	DDIT3	DNA damage inducible transcript 3	1649			■		■	■	■					
ENSG00000066336	SPI1	Spi-1 proto-oncogene	6688				■							■	
ENSG00000213281	NRAS	NRAS proto-oncogene, GTPase	4893				■		■	■					■
ENSG00000128340	RAC2	Rac family small GTPase 2	5880				■								■
ENSG00000115415	STAT1	signal transducer and activator of transcription 1	6772				■							■	
ENSG00000141510	TP53	tumor protein p53	7157				■	■	■	■	■				
ENSG00000124762	CDKN1A	cyclin dependent kinase inhibitor 1A	1026				■	■	■		■				
ENSG00000111276	CDKN1B	cyclin dependent kinase inhibitor 1B	1027				■	■	■		■				
ENSG00000100985	MMP9	matrix metallopeptidase 9	4318				■	■	■						
ENSG00000115414	FN1	fibronectin 1	2335				■								■
ENSG00000005961	ITGA2B	integrin subunit alpha 2b	3674				■								■
ENSG00000073756	PTGS2	prostaglandin-endoperoxide synthase 2	5743				■		■						
ENSG00000186350	RXRA	retinoid X receptor alpha	6256				■	■							
ENSG00000172115	CYCS	cytochrome c, somatic	54205				■			■					
ENSG00000169896	ITGAM	integrin subunit alpha M	3684					■							■
ENSG00000148516	ZEB1	zinc finger E-box binding homeobox 1	6935					■	■						
ENSG00000170458	CD14	CD14 molecule	929					■							■
ENSG00000140379	BCL2A1	BCL2 related protein A1	597					■		■					
ENSG00000143384	MCL1	MCL1 apoptosis regulator, BCL2 family member	4170						■	■					
ENSG00000185338	SOCS1	suppressor of cytokine signaling 1	8651						■					■	
ENSG00000185950	IRS2	insulin receptor substrate 2	866						■			■	■		

Supplementary File S4

KEGG pathways in cancer (hsa05200) DEGs

24 hub genes out of 34 DEGs detected in the KEGG Pathways in cancer (hsa05200) are shown in blue.

**ENSG00000130522**	**JUND**	**JunD proto-oncogene, AP-1 transcription factor subunit**	**Gene ID: 3727**
**ENSG00000177606**	**JUN**	**Jun proto-oncogene, AP-1 transcription factor subunit**	**Gene ID: 3725**
**ENSG00000170345**	**FOS**	**Fos proto-oncogene, AP-1 transcription factor subunit**	**Gene ID: 2353**
**ENSG00000136997**	**MYC**	**MYC proto-oncogene, bHLH transcription factor**	**Gene ID: 4609**
**ENSG00000066336**	**SPI1**	**Spi-1 proto-oncogene**	**Gene ID: 6688**
**ENSG00000213281**	**NRAS**	**NRAS proto-oncogene, GTPase**	**Gene ID: 4893**
ENSG00000144118	RALB	RAS like proto-oncogene B	Gene ID: 5899
ENSG00000017797	RALBP1	ralA binding protein 1	Gene ID: 10928
ENSG00000171777	RASGRP4	RAS guanyl releasing protein 4	Gene ID: 115727
**ENSG00000128340**	**RAC2**	**Rac family small GTPase 2**	**Gene ID: 5880**
ENSG00000100644	HIF1A	hypoxia inducible factor 1 subunit alpha	Gene ID: 3091
**ENSG00000100906**	**NFKBIA**	**NFKB inhibitor alpha**	**Gene ID: 4792**
**ENSG00000077150**	**NFKB2**	**nuclear factor kappa B subunit 2**	**Gene ID: 4791**
ENSG00000076604	TRAF4	TNF receptor associated factor 4	Gene ID: 9618
**ENSG00000115415**	**STAT1**	**signal transducer and activator of transcription 1**	**Gene ID: 6772**
**ENSG00000141510**	**TP53**	**tumor protein p53**	**Gene ID: 7157**
**ENSG00000124762**	**CDKN1A**	**cyclin dependent kinase inhibitor 1A**	**Gene ID: 1026**
**ENSG00000111276**	**CDKN1B**	**cyclin dependent kinase inhibitor 1B**	**Gene ID: 1027**
**ENSG00000145675**	**PIK3R1**	**phosphoinositide-3-kinase regulatory subunit 1**	**Gene ID: 5295**
**ENSG00000100985**	**MMP9**	**matrix metallopeptidase 9**	**Gene ID: 4318**
ENSG00000168036	CTNNB1	catenin beta 1	Gene ID: 1499
**ENSG00000115414**	**FN1**	**fibronectin 1**	**Gene ID: 2335**
**ENSG00000005961**	**ITGA2B**	**integrin subunit alpha 2b**	**Gene ID: 3674**
ENSG00000125384	PTGER2	prostaglandin E receptor 2	Gene ID: 5732
ENSG00000171522	PTGER4	prostaglandin E receptor 4	Gene ID: 5734
**ENSG00000073756**	**PTGS2**	**prostaglandin-endoperoxide synthase 2**	**Gene ID: 5743**
ENSG00000139679	LPAR6	lysophosphatidic acid receptor 6	Gene ID: 10161
**ENSG00000186350**	**RXRA**	**retinoid X receptor alpha**	**Gene ID: 6256**
**ENSG00000182578**	**CSF1R**	**colony stimulating factor 1 receptor**	**Gene ID: 1436**
**ENSG00000119535**	**CSF3R**	**colony stimulating factor 3 receptor**	**Gene ID: 1441**
**ENSG00000169429**	**CXCL8**	**C-X-C motif chemokine ligand 8**	**Gene ID: 3576**
**ENSG00000121966**	**CXCR4**	**C-X-C motif chemokine receptor 4**	**Gene ID: 7852**
**ENSG00000172115**	**CYCS**	**cytochrome c, somatic**	**Gene ID: 54205**
ENSG00000117394	SLC2A1	solute carrier family 2 member 1	Gene ID: 6513

Supplementary File S5

SMG_DI_draft_31.07.2026_text_only_UPDATED.docx

Karolinska Institute

Document Details

Submission ID

trn:oid:::3117:611427219

Submission Date

Jul 31, 2026, 1:04 AM GMT+2

Download Date

Jul 31, 2026, 1:06 AM GMT+2

File Name

SMG_DI_draft_31.07.2026_text_only_UPDATED.docx

File Size

68.6 KB

11 Pages

5,355 Words

35,737 Characters

0% Overall Similarity

The combined total of all matches, including overlapping sources, for each database.

Match Groups

0 Not Cited or Quoted 0%
Matches with neither in-text citation nor quotation marks

0 Missing Quotations 0%
Matches that are still very similar to source material

0 Missing Citation 0%
Matches that have quotation marks, but no in-text citation

0 Cited and Quoted 0%
Matches with in-text citation present, but no quotation marks

Top Sources

0% Internet sources

0% Publications

0% Submitted works (Student Papers)

Integrity Flags

1 Integrity Flag for Review

Replaced Characters
84 suspect characters on 6 pages
Letters are swapped with similar characters from another alphabet.

Our system's algorithms look deeply at a document for any inconsistencies that would set it apart from a normal submission. If we notice something strange, we flag it for you to review.

A Flag is not necessarily an indicator of a problem. However, we'd recommend you focus your attention there for further review.

Match Groups

- **0** Not Cited or Quoted 0%
  Matches with neither in-text citation nor quotation marks
- **0** Missing Quotations 0%
  Matches that are still very similar to source material
- **0** Missing Citation 0%
  Matches that have quotation marks, but no in-text citation
- **0** Cited and Quoted 0%
  Matches with in-text citation present, but no quotation marks

Top Sources

0% Internet sources
0% Publications
0% Submitted works (Student Papers)

T cell transcriptomes in microgravity *in vivo*

Abstract

Background: The health of astronauts and success of space missions are contingent on resilience to the cumulative stressors of the space environment, including radiation, isolation, confinement and microgravity. While these factors are known to disrupt physiological function at molecular and cellular levels, the isolated impact of microgravity itself remains poorly defined. **Objective:** To explore in vivo effects of long-term simulated microgravity (SMG) on T cell transcriptomes by analyzing gene expression changes in peripheral blood T cells of healthy volunteers. **Methods:** A three-week SMG exposure was completed by ten healthy male participants, during which peripheral blood specimens were harvested at five critical junctures (covering baseline, intra-exposure, and recovery stages). Post-collection, T lymphocytes were enriched, and their total RNA was extracted. Following next-generation sequencing, a rigorous bioinformatics framework was employed to analyze and interpret the volunteers' global gene expression dynamics. **Results:** Ground-based SMG induces widespread deregulation of signal-transduction cascades and gene-regulatory circuitry within T lymphocytes. SMG influences several key cell signaling pathways in vivo including those involved in cell proliferation, cell cycle regulation, maintaining genome integrity, as well as oncogenic and tumor suppressors cell signaling pathways. Corresponding hub genes found to be differentially expressed in majority of tested healthy volunteers. **Conclusion:** Important intracellular signaling cascades and gene regulatory networks within human T cells are perturbed by SMG *in vivo.* We conclude that microgravity deregulates cellular signaling, leading to widespread alterations in gene expression networks. Our findings indicate that the mechanical unloading imposed by SMG, even at a level that induces only mild systemic effects, is sufficient to upset the equilibrium of core signaling pathways and alter associated gene regulatory networks.

Keywords: T cell, gene expression, transcriptome, microgravity, signaling pathway, gene network

iThenticate Page 5 of 14 - Integrity Submission Submission ID trn:oid:::3117:611427219

1. Introduction

The spaceflight environment presents a complex spectrum of physiological stressors that profoundly differ from conditions on Earth, posing significant risks to astronaut health and mission success. NASA has categorized these into five primary hazards: isolation, confinement, distance from Earth, gravity alterations, and exposure to space radiation [1, 2]. Furthermore, additional factors such as hypomagnetic environment, circadian rhythm disruption, and perturbations in the environmental microbiome can further compound these challenges and influence crew wellbeing [3]. Among these stressors, the effects of Galactic Cosmic Radiation (GCR) on biomolecular and cellular processes have been extensively characterized through both spaceflight and ground-based simulation studies [4, 5]. In contrast, the specific contribution of microgravity to cellular dysfunction, particularly in immune cell signaling, proliferation, and metabolism, remains insufficiently defined and a subject of active investigation [6]. Recent evidences suggest that MG may be a dominant driver of the physiological stress observed in spaceflight, significantly impacting biological systems [7, 8].

To delineate the specific *in vivo* impacts of microgravity on human T lymphocytes, we applied long-term SMG to healthy volunteers. Our analysis of T cell transcriptomes revealed significant deregulation of core gene networks and oncogenic signaling pathways. A critical and novel finding was the observation of substantial inter-individual heterogeneity, with several distinct types of transcriptional responses identified among the volunteers. This evidence compellingly supports a shift in perspective: rather than pursuing a universal solution, effective countermeasures may require a personalized medicine approach that accounts for individual variability in response to microgravity.

A microgravity investigation, conducted on a cohort of ten healthy individuals, was designed to determine whether the absence of gravity alone elicits detectable changes of T cell transcriptomes in vivo. Our findings could not only shed light on fundamental mechanisms of space cell biology but

also serve as a pivotal stepping stone toward developing targeted strategies to safeguard human health during extended stays in microgravity.

Figure 1. Procedural setup for the ground-based SMG study.

The time course of the SMG experiment and sample processing flow chart.

2. Methods

2.1. *Simulated microgravity experiment setup*

Ten healthy male participants, aged 24 to 32 years, participated in a three-week simulated microgravity (SMG) experiment using dry immersion, with no countermeasures administered, as outlined in [7]. All volunteers gave their written consent prior to the study, adhering to the ethical requirements of the Helsinki Code of Medical Ethics regarding Human Specimens.

2.2. *Peripheral blood sample collection*

During the SMG protocol, peripheral blood samples (10 mL each) were collected from every volunteer into sodium heparin tubes at five predefined time points: 7 days prior to SMG onset (day –7; background, bg), at 7, 14, 21 days of microgravity exposure, and 7 days after the end of SMG exposure (day 28) (Figure 1).

2.3. *CD3+ T cell isolation*

Ficoll-Paque™ PLUS (Cytiva) was employed to obtain PBMCs. CD3+ T-cell subset was then positively selected (Stem Cell Technology), resulting in a typical purity of 95%.

2.4. *RNA Extraction and Sequencing*

iThenticate Page 5 of 14 - Integrity Submission Submission ID trn:oid:::3117:611427219

iThenticate Page 6 of 14 - Integrity Submission Submission ID trn:oid:::3117:611427219

RNA was extracted, quantified, and RNA quality was assessed via AGE in 1% UltraPure Agarose (Cat. #16500500; Invitrogen, Waltham, MA, USA) as described previously [7]. Gel imaging conducted on a ImageQuant LAS 4000 imaging system as described [9]. Following successful completion of both internal and external quality control assessments (Novogene, Hong Kong), the RNA libraries were processed to 150bp paired-end PE sequencing (Illumina HiSeq; Novogene), followed by standard data quality control procedures.

2.5. *RNA-Seq Bioinformatics*

Sequencing reads mapped to GRCh38/hg38 genome assembly employing HISAT2 (v.2.0.5). Initial bioinformatic preprocessing (quality filtering, alignment, and GO term enrichment) was conducted by Novogene (Hong Kong). Differential gene expression was assessed using the DESeq2 framework within R. To identify expression patterns, log_2-transformed ratios were clustered via hierarchical clustering, K-means partitioning, and self-organizing maps (SOM). PCA and heatmaps were generated in NASQAR (NYUAD). Functional annotation of genes associated with the identified peaks *was carried out employing* the NCBI Gene portal as detailed previously [6, 10, 11].

3. Results

3.1. *RNA-seq and gene expression data*

A total of fifty samples (10 volunteers × 5 time points) yielded up to 58,676 transcripts expression data, which were assigned to over 200 cell signaling pathways. RNA-seq dataset is publicly accessible via the NCBI GEO database under accession number.

Differential expression analysis was performed for T cell samples at each time point, with background expression values derived from the pre-immersion baseline (−7d). Transcript levels of differentially expressed genes (DEGs) were visualized on volcano plots of −log10 (q-value) vs. log2(FC) for each time point (Supplementary File 1).

3.2. *Gene networks and pathways*

To identify the most prominently affected cell signaling networks, twelve KEGG pathways with the highest statistical significance (lowest corrected p-values) were selected from the complete pathway annotation list (Table 1; Supplementary File 2).

From the DEGs mapping to these twelve top-ranked pathways, 44 interconnected genes that appeared in at least two of the selected pathways were designated as hub genes (Supplementary File 3). These genes were subsequently subjected to detailed comparative expression analysis across all time points (Table 2).

3.3. *Cancer genes expression patterns and oncogenic pathways*

A panel of DEGs was mapped to three cancer-related KEGG pathways that were among the top twelve most statistically significant pathways: hsa05200, hsa05206, and hsa05202.

Among the 34 detected DEGs mapping to the KEGG Pathways in cancer (hsa05200), 24 genes qualified as hub genes based on their presence in at least two of the top twelve pathways. (Supplementary File 4). Non-normalized expression values for each of the 24 hub genes were analyzed individually for each of the 10 volunteers across all time points (Figure 2) with the aligned expression patterns.

Figure 2. Expression of 24 hub genes in KEGG Pathways in cancer (hsa05200).
Non-normalized expression values (Log2 fold change) of detected hub genes were analyzed for each of 10 volunteers (Vol.#1-10) across all time points, and the aligned patterns are shown as a heatmaps.

3.4. *Gene co-expression*

Gene co-expression data were analyzed for each volunteer across all time points, and the results are shown as individual Venn diagrams for each of the 10 subjects (Figure 3). Each diagram's petals quantify time-point-specific genes, with overlaps denoting shared genes across two or more time points.

iThenticate Page 6 of 14 - Integrity Submission Submission ID trn:oid:::3117:611427219

iThenticate Page 7 of 14 - Integrity Submission Submission ID trn:oid:::3117:611427219

Figure 3. Co-expression Venn diagrams of differentially expressed genes across five time points for each volunteer. Petals: unique gene counts per time point. Overlaps: gene sets shared between ≥2 time points. The sum of the numbers within each ellipse represents the total number of DEGs detected at that time point.

3.5. *Principal component analysis (PCA)*

PCA of the T cell transcriptomic data revealed that samples clustered primarily by volunteer identity rather than by exposure stage across all time points. Volunteers 3–8 consistently formed the main central cluster, whereas Volunteer 9 occupied a distinct and highly separated region of the PCA space throughout the study; Volunteers 1, 2, and 10 were also positioned outside the main cluster but remained comparatively closer to it. Despite this pronounced stratification by individual, coordinated shifts in PCA coordinates were observed between baseline (-7 days) and the immersion period (7–21 days) for all participants. The most pronounced displacements occurred at 14 and 21 days of exposure, while the 28-day samples partially reverted toward their respective pre-immersion positions (Figure 4).

Figure 4. Group Principal Component Analysis (PCA). PCA of transcriptomic profiles across the SMG exposure time course, with each volunteer's trajectory revealing pronounced inter-individual stratification and heterogeneous response dynamics.

iThenticate Page 7 of 14 - Integrity Submission Submission ID trn:oid:::3117:611427219

4. **Discussion**

4.1. Cytokine-cytokine receptor interaction

The identification of ten genes (IL1B, IL7R, CXCR4, CXCL8, CSF1R, CSF2RB, CSF3R, TNF, TNFRSF1A, TNFSF10) within the KEGG Cytokine-cytokine receptor interaction pathway (hsa04060) reveals a coordinated perturbation of ligand-receptor signaling networks governing inflammation, leukocyte trafficking, and hematopoietic regulation. Microgravity consistently dysregulates cytokine networks and immune cell function, altering expression of pro-inflammatory IL-1β and TNF while shifting inflammatory set points that compromise host defense [12-16]. Recent single-cell analyses have further confirmed that immune suppression signatures are invariant whether evoked by SMG or orbital microgravity, reinforcing the robustness of these findings [17]. Chemokine components CXCL8 and CXCR4 likely mediate microgravity-sensitive changes in neutrophil and lymphocyte chemotaxis and tissue homing, mechanisms repeatedly disrupted in both real and SMG experiments [13, 15]. Colony stimulating factor receptors (CSF1R, CSF2RB, CSF3R) and IL7R directly link to altered myeloid and lymphoid differentiation and hematopoietic output observed in spaceflight studies [13, 16]. TNF, TNFRSF1A, and TNFSF10 highlight plausible routes through which microgravity may modulate apoptosis, survival signaling, and inflammatory amplification via receptor-mediated cascades, providing a molecular framework connecting transcriptional findings to immune and hematopoietic phenotypes documented in experimental literature [12, 14, 18]. The involvement of NF-κB-mediated transcription further underscores the gravity-sensitive nature of these inflammatory pathways [19].

4.2. T cell receptor signaling

Eight genes (ICOS, JUN, FOS, MAP3K8, NFKBIA, NFKB2, TNF, PIK3R1) mapping to the T cell receptor signaling pathway (hsa04660) indicate coordinated disruption of antigen receptor-proximal signaling, co-stimulation, transcriptional activation, and downstream survival programs under microgravity. TCR engagement normally recruits MAP kinases and NF-κB while activating AP-1 (JUN/FOS) to drive cytokine production and proliferation; perturbation of these nodes blunts T cell activation and effector differentiation, consistent with gravity-sensitive signaling in lymphocytes [17, 20]. Gravitational mechanosignals governing T-cell activation have been molecularly defined, with early studies demonstrating that microgravity inhibits immediate early gene transcription through disruption of the Rel/NF-κB pathway [19, 21]. PI3K regulatory subunits such as PIK3R1 function as central hubs linking mechanotransduction to metabolic and trafficking responses in T cells, offering a mechanistic route through which altered mechanical forces reprogram activation thresholds and migratory behavior [18, 22, 23]. Exposing primary human T cells to both transient and prolonged gravitational unloading, via parabolic flight and clinorotation, respectively, has confirmed the sensitivity of these pathways to altered gravity [24].

Co-stimulatory molecules like ICOS and MAP3K8 further shape TCR signaling amplitude, while changes in NF-κB components (NFKB2, NFKBIA) and TNF can shift the balance between proliferation, survival, and apoptosis under stress [20, 21]. Collectively, these genes suggest that microgravity impairs adaptive immunity by simultaneously dampening co-stimulatory input, rewiring MAPK/PI3K mechanosensitive signaling, and altering NF-κB/AP-1 transcriptional programs essential for robust T cell responses.

4.3. MAPK signaling pathway

Ten MAPK pathway genes (JUND, JUN, FOS, MYC, GADD45A, GADD45B, DDIT3, MAP3K8, IL1B, TNF) mapping to hsa04010 point to a gravity-sensitive network coupling stress sensing, transcriptional reprogramming, and cell fate decisions. MAP3K8 and related MAP3Ks occupy upstream nodes determining ERK/JNK/p38 activation patterns; systematic analyses show combinatorial MAPK activation can switch outcomes between proliferation and apoptosis, an effect plausibly translating into altered tissue maintenance under microgravity [25]. Early work demonstrated that coordinated signaling via the MAPK network and SRC-kinase is required for the mechano-induced expression of the immediate-early gene EGR-1 and the pro-inflammatory mediator NF-κB, establishing the mechanosensitivity of this pathway [26]. AP-1

iThenticate Page 9 of 14 - Integrity Submission Submission ID trn:oid:::3117:611427219

components (JUN/FOS/JUND) and MYC act as immediate early transcriptional effectors of MAPK signaling, controlling cell cycle entry, metabolic reprogramming, and survival—programs modulated by mechanical unloading and environmental stressors [27]. Studies in human bone marrow mesenchymal stem cells have confirmed that SMG inhibits cell proliferation and activates MAPK pathway components [28]. Stress-responsive genes including GADD45A/B and DDIT3 integrate DNA damage and ER stress signals with MAPK outputs, providing mechanistic routes to growth arrest and pro-apoptotic phenotypes reported in cells exposed to SMG [29]. Inflammatory mediators IL1B and TNF within this MAPK module links mechanical perturbation to cytokine-driven amplification loops that reshape MAPK dynamics and downstream transcriptional programs [30]. These data support a model wherein microgravity perturbs MAPK network topology and timing via MAP3K nodes, AP-1/MYC transcriptional control, and stress-responsive effectors, thereby altering proliferation, survival, and inflammatory outputs consistent with experimental observations [29].

4.4. Pathways in cancer

The 23 genes mapping to Pathways in cancer (hsa05200) including JUN, FOS, MYC, SPI1, NRAS, RAC2, NFKBIA, NFKB2, STAT1, TP53, CDKN1A, CDKN1B, PIK3R1, MMP9, FN1, ITGA2B, PTGS2, RXRA, CSF1R, CSF3R, CXCL8, CXCR4, CYCS suggest microgravity engages a cancer-relevant network coordinating cell cycle control, stress responses, survival/apoptosis decisions, cytoskeletal and extracellular matrix remodeling, and signaling through RAS/PI3K and inflammatory axes. Alterations in TP53, CDKN1A/B, MYC, and AP-1 components can reprogram proliferation and senescence programs, while changes in CYCS and TNF/IL1 mediators bias cells toward apoptosis or survival depending on context. Recent work demonstrates microgravity perturbs the p53 regulatory network and aging-associated targets, linking altered gravity to DNA damage and stress signaling converging on tumor suppressor pathways [7, 31]. Studies using colorectal cancer organoids have demonstrated that SMG significantly affects tumor growth patterns and drug responses, providing functional validation of these pathway perturbations [32]. Concurrent modulation of NRAS, RAC2, and PIK3R1 indicates rewiring of proliferative and motility signaling that alters cytoskeletal dynamics and promotes three-dimensional spheroid formation or altered adhesion phenotypes observed in microgravity cancer models, with downstream consequences for drug sensitivity and metabolic state through MYC-dependent programs [33–35]. The potential of using space-based investigations to inform cancer research on Earth has been increasingly recognized, with microgravity revealing fundamental aspects of tumor biology [36]. Matrix remodeling genes (MMP9, FN1, ITGA2B) together with chemokine/chemokine receptor pairs (CXCL8/CXCR4) provide mechanistic basis for shifts in invasion, migration, and immune cell interactions under unloading conditions, potentially unmasking oncogenic behaviors in vitro while altering chemotherapy responses [34, 35, 37]. Enrichment of cancer pathway genes in our microgravity signature argues that altered gravitational forces reconfigure core regulatory circuits governing proliferation, stress adaptation, and cell-matrix interactions—changes directly relevant to tissue homeostasis, regenerative capacity, and interpretation of cancer cell phenotypes in space biology studies [7, 31, 38].

4.4.1. Transcriptional misregulation in cancer

Fourteen genes mapped to Transcriptional misregulation in cancer (hsa05202): ITGAM, ZEB1, RXRA, CSF1R, CD14, TP53, MMP9, SPI1, CXCL8, CDKN1A, CDKN1B, MYC, BCL2A1, DDIT3 point to a gravity-sensitive axis wherein altered mechanical input transduces into persistent changes in transcriptional programs governing inflammation, epithelial-mesenchymal plasticity, cell cycle control, and survival. Transcription factors and co-regulators including ZEB1, MYC, and SPI1 can rewire lineage and stress response programs, while modulators of chromatin and nuclear signaling (RXRA) and stress-induced effectors (DDIT3/CHOP, BCL2A1) determine whether cells adopt adaptive, senescent, or apoptotic fates. Recent syntheses of microgravity transcriptomics emphasize such transcriptional reprogramming as a hallmark of unloading underlying many downstream phenotypes [39, 40]. Studies on intestinal stem cells and epithelial regeneration have further highlighted the impact of microgravity on tissue-specific transcriptional programs [41]. Innate immune and myeloid regulators (CD14, CSF1R, ITGAM) together with inflammatory mediators (CXCL8, MMP9)

iThenticate Page 9 of 14 - Integrity Submission Submission ID trn:oid:::3117:611427219

iThenticate Page 10 of 14 - Integrity Submission Submission ID trn:oid:::3117:611427219

provide a direct molecular link between transcriptional misregulation and altered immune-stromal crosstalk in microgravity, consistent with reports that gene regulatory networks controlling inflammation and extracellular matrix remodeling are particularly labile under real and simulated microgravity [40, 42]. SMG has been shown to inhibit osteogenic differentiation through downregulation of ZEB1, demonstrating the functional relevance of this transcription factor in unloading responses [43]. Perturbation of tumor suppressor and cell cycle checkpoints (TP53, CDKN1A, CDKN1B) combined with changes in adhesion and matrix remodeling genes explains frequently observed shifts in proliferation, three-dimensional growth, and stressor resistance/sensitivity in microgravity models [44]. Mechanistic work on ZEB1 further demonstrates how EMT-linked transcription factors can promote replication stress tolerance while creating vulnerabilities in DNA damage responses—a duality plausibly operating in unloaded cells to shape long-term tissue outcomes [39, 42]. These observations support a model wherein microgravity drives durable transcriptional misregulation across immune, stress, and developmental programs via nodes identified in hsa05202, linking altered mechanics to changes in tissue remodeling, immune competence, and cell fate decisions central to organismal adaptation during spaceflight.

4.4.2. MicroRNAs in cancer pathways

The mapping of MYC, NRAS, TP53, DDIT3, MCL1, ZEB1, SOCS1, CDKN1A, CDKN1B, IRS2, MMP9, and PTGS2 to the MicroRNAs in cancer pathway (hsa05206) highlights microRNAs as pivotal post-transcriptional regulators that can amplify or buffer microgravity-induced transcriptional shifts, reshaping cell fate, stress responses, and signaling plasticity. MicroRNAs control many observed genes—miR-34/miR-145 families intersect with MYC and TP53 networks, miR-21 and miR-155 modulate inflammatory and survival nodes including MCL1 and SOCS1, and miR-200 family members regulate EMT drivers such as ZEB1, suggesting gravity-dependent changes in miRNA expression can produce coordinated downstream effects on proliferation, apoptosis, EMT, and metabolic signaling [45]. Integrated peripheral-lymphocytic microRNA/mRNA transcriptome co-profiling of astronauts has revealed consistent dysregulation patterns following spaceflight [46]. Recent omics studies of tumor cells and broader microgravity transcriptomic surveys report consistent dysregulation of miRNA networks under real and simulated microgravity, with consequent alterations in cell cycle regulators (CDKN1A/B), pro-apoptotic effectors (DDIT3), and matrix remodeling enzymes (MMP9), collectively influencing spheroid formation, drug sensitivity, and stress tolerance in unloaded cells [39, 47]. MicroRNA expression profiling in SMG-treated human colon cancer cells has identified specific miRNA-mRNA regulatory networks that mediate adaptive responses [48]. These findings suggest a model wherein microgravity perturbs miRNA biogenesis or stability, modulating abundance and activity of key oncogenic and tumor suppressor targets (MYC, NRAS, TP53, IRS2), with this post-transcriptional layer constituting a major determinant of phenotypic plasticity observed in space biology experiments [49]. Integrating miRNA profiling with gene-level signatures will be essential to resolve causal regulatory chains and identify miRNA-based biomarkers or countermeasures mitigating microgravity-induced dysregulation of proliferation, survival, and immune-related pathways.

4.5. Apoptosis pathway

Seventeen genes mapped to the Apoptosis pathway (hsa04210): JUN, FOS, NRAS, NFKBIA, TNF, TNFRSF1A, TNFSF10, CYCS, GADD45A/B/G, DDIT3, MCL1, CSF2RB, PIK3R1, TP53, BCL2A1 define a multilayered program through which microgravity can tip the balance between survival and programmed cell death via mitochondrial, death receptor, ER stress, and pro-survival kinase axes. Mitochondrial apoptosis, centrally mediated by cytochrome c release and BCL-2 family regulation (CYCS, MCL1, BCL2A1), exhibits sensitivity to redox changes and mechanical stress, with repeated implication in microgravity-enhanced apoptosis across cell types [50–53]. Recent studies have identified PIEZO1 as a mediator of endothelial cell apoptosis through HMGA2 under SMG, revealing novel mechanotransductive links to cell death [54]. p53 and stress-responsive effectors (TP53, GADD45 family, DDIT3/CHOP) integrate DNA damage and ER stress signals to promote either repair or mitochondrial death pathways; recent mechanistic reviews highlight how altered gravity amplifies these stress signals and mitochondrial vulnerability [52, 55]. Studies in vascular endothelial cells and

iThenticate Page 10 of 14 - Integrity Submission Submission ID trn:oid:::3117:611427219

lymphoblastoid cells have confirmed that SMG enhances apoptosis through the mitochondrial pathway [56, 57]. Concurrently, death receptor signaling (TNF, TNFRSF1A, TNFSF10) and NF-κB modulators (NFKBIA) provide extracellular and transcriptional inputs that can either reinforce apoptosis or engage compensatory survival programs. PI3K regulatory subunits (PIK3R1) and CSF2RB link cytokine-mediated trophic signaling to AKT/ERK survival cascades, offering routes through which microgravity-induced cytokine dysregulation secondarily modulates apoptotic thresholds. Empirical studies in real and simulated microgravity report increased caspase activation, mitochondrial outer membrane permeabilization, and ER stress markers consistent with this network-level model, indicating that coordinated perturbation of mitochondrial effectors, death receptor ligands, stress sensors, and pro-survival kinases captured by hsa04210 plausibly underlies cell type- and context-dependent apoptosis phenotypes observed during unloading [50–53].

4.6. Cell cycle regulation pathway

Seven cell cycle genes (CDKN1A, CDKN1B, GADD45A, GADD45B, GADD45G, MYC, TP53) mapping cleanly to the KEGG Cell cycle pathway (hsa04110) point to coordinated disruption of G1/S checkpoint control, DNA damage sensing, and replication stress responses in unloaded conditions. Elevated expression of cyclin-dependent kinase inhibitor CDKN1A/p21 has been repeatedly observed in bone and other mechanically sensitive tissues during spaceflight and SMG, where increased p21 correlates with cell cycle arrest, reduced proliferation, and impaired regenerative capacity. Genetic or pharmacologic modulation of p21 alters these outcomes in rodent flight models, directly linking CDKN1A dynamics to gravity-dependent tissue maintenance [7, 52, 58, 59]. NASA experiments have specifically investigated the role of CDKN1a/p21 in mesenchymal stem cell proliferation and regeneration under microgravity conditions [60]. Studies in human bone marrow mesenchymal stem cells have confirmed that SMG inhibits proliferation by inducing G1 phase arrest through cell cycle regulator modulation [61]. Stress-inducible GADD45 family members act as sensors coupling DNA damage and environmental stress to cell cycle checkpoints and p38/MAPK signaling; their induction in microgravity can enforce growth arrest or facilitate repair depending on context [58, 62]. The interplay between TP53 and MYC further determines whether cells enter transient arrest, senescence, or apoptotic programs: microgravity-associated perturbations in p53 signaling and MYC-driven metabolic programs have been reported in recent transcriptomic surveys, suggesting altered gravitational loading reprograms the balance between proliferation and protective arrest [7, 33]. Together, these nodes provide mechanistic explanation for frequently observed reductions in proliferation, altered differentiation trajectories, and increased replication stress in cells exposed to real or simulated microgravity, identifying CDKN1A/p21, GADD45s, TP53, and MYC as candidate molecular levers for countermeasures aimed at preserving tissue regenerative potential during long-duration missions.

4.7. FoxO signaling pathway

The presence of IL7R, IRS2, and TNFSF10 in our microgravity signature and their mapping to the FoxO signaling pathway (hsa04068) point to a conserved stress-responsive axis through which altered gravitational loading can reprogram cell survival, metabolism, and immune homeostasis. FoxO transcription factors integrate upstream growth factor and insulin/PI3K-AKT inputs—with IRS2 serving as canonical adaptor linking insulin/IGF signaling to AKT and consequently to FoxO phosphorylation and nuclear exclusion—so changes in IRS2 expression or activity under microgravity could shift FoxO localization and downstream programs controlling oxidative stress defenses, autophagy, and metabolic reprogramming [63]. IL7R signaling intersects with FoxO-regulated lymphoid survival and homeostatic proliferation pathways; altered IL-7R dynamics in unloaded conditions may therefore perturb FoxO-dependent maintenance of T and B cell pools [64, 65]. TNFSF10 (TRAIL) engages extrinsic apoptotic pathways modulated by FoxO targets providing a route through which FoxO activity tunes the balance between adaptive stress responses and programmed cell death in microgravity [66]. The broader roles of FOXOs in acute and chronic neurological disorders further underscore their importance as stress-responsive transcription factors [67]. Collectively, these connections suggest microgravity-induced shifts in growth factor signaling (IRS2), cytokine receptor input (IL7R), and death ligand activity (TNFSF10)

iThenticate Page 12 of 14 - Integrity Submission Submission ID trn:oid:::3117:611427219

converge on FoxO transcriptional control to alter redox homeostasis, cell cycle arrest, autophagy, and apoptosis—mechanisms highlighted in studies linking cytokine and insulin-related signaling to FoxO-mediated homeostasis [63, 65, 68].

4.8. Primary immunodeficiency

The detection of ICOS, IL7R, and BTK within the Primary immunodeficiency pathway (hsa05340) suggests microgravity impinges on core nodes required for lymphocyte development, co-stimulation, and B cell receptor signaling, potentially producing phenotypes that phenocopy aspects of primary immunodeficiency. IL7R is essential for lymphoid progenitor survival and B/T lineage differentiation; altered IL7R signaling under microgravity could reduce lymphopoiesis and repertoire diversity, as demonstrated in human B cell progenitors where IL-7R activity drives expansion and V(D)J usage patterns [64]. BTK serves as a central transducer of B cell receptor signals required for maturation and antibody responses; perturbation of BTK expression or activity in altered gravity would likely impair humoral immunity and vaccine responsiveness [69]. ICOS provides critical co-stimulatory input for T follicular helper cell function and germinal center responses, so its dysregulation could further compromise B-T cell collaboration and long-lived antibody production [70]. Spaceflight studies have demonstrated persistent alterations in adaptive immunity, including impaired vaccine responses and altered T-cell differentiation [71, 72]. Monitoring studies of T-cell activation and differentiation in microgravity have confirmed the sensitivity of these developmental pathways to gravitational unloading [73]. Together, these three genes provide a mechanistic link between microgravity-induced transcriptional changes and clinically relevant reductions in adaptive immune competence reported in spaceflight and SMG studies [13, 64, 69].

4.9. Osteoclast differentiation

Fourteen genes mapped to Osteoclast differentiation (hsa04380): IL1B, CSF1R, JUND, JUN, FOS, SPI1, SOCS1, NFKBIA, NFKB2, STAT1, TNF, TNFRSF1A, PIK3R1, BTK define a coherent signaling module linking inflammatory cues, receptor tyrosine/kinase inputs, transcriptional effectors, and NF-κB/PI3K survival programs to the generation and activation of bone-resorbing osteoclasts. Colony stimulating factor signaling through CSF1R and pro-inflammatory ligands including TNF and IL1B are established drivers of osteoclast lineage commitment and fusion; in microgravity these cytokine axes are frequently dysregulated, providing molecular explanation for enhanced osteoclastogenesis and accelerated bone resorption observed in unloaded models and astronauts [74–76]. Spaceflight studies have directly demonstrated that microgravity affects osteoclastogenesis by increasing the RANKL/OPG ratio [77]. Downstream, MAPK AP-1 components (JUN, FOS, JUND) and NF-κB regulators (NFKBIA, NFKB2) cooperate with lineage transcription factors such as SPI1/PU.1 to induce osteoclast-specific programs, while PIK3R1 and BTK integrate trophic and receptor signals to modulate survival, cytoskeletal reorganization, and resorptive activity [69, 78]. Systems biology analyses have revealed coordinated osteoclast and osteoblast responses to modeled microgravity [79]. Negative feedback modulators like SOCS1 and STAT-dependent signaling tune differentiation magnitude and duration; perturbation of these checks in microgravity can shift balance toward excessive resorption [18, 76]. Enrichment of these genes in hsa04380 supports a model wherein microgravity-induced cytokine imbalance, altered CSF1R/BTK/PI3K signaling, and reprogrammed AP-1/NF-κB transcriptional responses converge to promote osteoclast differentiation and activity—mechanisms aligning with documented microgravity-associated bone loss and osteoclastogenic pathways [52, 74, 80].

4.10. Regulation of actin cytoskeleton

The Regulation of actin cytoskeleton (hsa04810) enrichment driven by ITGAM, ITGA2B, CD14, FN1, RAC2, NRAS, and PIK3R1, provides a mechanistic bridge between altered gravitational loading and the rapid, cell type-specific reorganization of adhesion, force transmission, and motility characterizing microgravity responses. Integrin-ECM complexes (ITGA2B/ITGAM; FN1) form the primary mechanical linkage to intracellular actin networks and focal adhesions; disruption of this reciprocity under unloading remodels focal adhesion

iThenticate Page 12 of 14 - Integrity Submission Submission ID trn:oid:::3117:611427219

composition and signaling through RAS/PI3K and Rho family GTPases, producing downstream changes in lamellipodial dynamics, phagocytic cup formation, and cell spreading repeatedly observed in immune and stromal cells in SMG [9, 81–84]. The cytoskeleton, acting through mechanosensitive channels, is now recognized as a sensor of gravitational fluctuations and a primary mechanotransductive mechanism [85]. RAC2 and NRAS coordinate actin nucleation and membrane protrusion; genetic and functional studies demonstrate Rac1/Rac2 essential for immune cell motility and antigen responses, linking RAC2 signal to impaired chemotaxis and altered immune function reported in unloading conditions [10, 11, 57]. PIK3R1 couples integrin and growth factor receptors to actin remodeling effectors (WASP/WAVE, ARP2/3) and metabolic programs sustaining cytoskeletal turnover, offering a plausible route through which changes in PI3K regulatory subunits translate mechanical cues into persistent transcriptional and phenotypic shifts [22, 82, 83]. Nuclear actin regulators and actin-linked transcriptional co-factors further connect cytoskeletal remodeling to gene expression: *nuclear WASP protein modulates T lymphocyte* transcriptional programs, illustrating how actin regulators directly influence lineage-specific transcriptional outputs relevant to our immune cell signatures [9]. RhoGTPases have been identified as key players in mechanotransduction during microgravity exposure, linking cytoskeletal dynamics to downstream signaling [86]. Comparative transcriptomic work demonstrates responses to SMG are distinct from hydrodynamic stress yet converge on cytoskeletal and adhesion modules, underscoring actin regulatory network as a reproducible gravity-sensitive hub across experimental systems [10, 11, 87, 88]. Dynamic gene expression response studies in human T cells have further confirmed the centrality of cytoskeletal regulation in gravity sensing [6, 7, 10, 11, 87, 88]. Recent bioinformatic and experimental analyses show microgravity-induced cytoskeletal instability correlates with altered focal adhesion signaling, changes in RAC-dependent motility, and emergence of three-dimensional aggregation or spheroid phenotypes—outcomes mechanistically consistent with the identified gene set, with implications for immune competence, wound healing, and tissue architecture during long-duration missions [83, 85, 87]. The cytoskeleton and its role in T cell migration and activation under microgravity conditions has been extensively characterized, providing functional validation of these pathway perturbations [89]. Together, these data support a model wherein integrin-ECM disruption, RAS/PI3K-Rac signaling, and actin-nuclear coupling form a coherent mechanotransductive axis translating reduced gravitational force into altered cell behavior and durable transcriptional reprogramming [10, 11, 88].

4.11. *PCA-based inter-individual stratification and heterogeneous SMG response trajectories*

The PCA trajectories suggest a strong inter-individual effect that is substantially larger than the group-level effect of SMG. At all time points, samples cluster primarily according to volunteer identity rather than according to exposure stage, indicating that baseline transcriptional differences between individuals remain a dominant source of variance. Specifically, volunteers 3-8 consistently formed the main cluster, whereas volunteer 9 remains highly separated and occupies a distinct region of PCA space throughout the study. Volunteers 1, 2 and 10 are also distinct but somewhat closer to the main cluster. Despite this pronounced stratification, a temporal response to SMG is still apparent. Between baseline (-7 days) and exposure (7-21 days), several individuals display coordinated shifts in PCA space, suggesting a common physiological adaptation. The movement is most pronounced during immersion (particularly at 14 and 21 days), whereas at 28 days the samples partially return toward their initial positions, consistent with recovery following cessation of the intervention. However, the magnitude and direction of these trajectories differ among volunteers, indicating substantial heterogeneity in response. Overall, the PCA analysis supports the conclusion that SMG induces a detectable transcriptomic effect, but that the response is strongly modulated by individual-specific factors, leading to marked stratification of the cohort and variable adaptation dynamics between volunteers.

These findings are consistent with human bed-rest transcriptomic studies, which demonstrate that while a core set of genes responds consistently to unloading, baseline expression profiles are highly individual-specific and remain the primary determinant of variance throughout the intervention [90]. This observation aligns with the broader framework proposed for space medicine, wherein omics-based analyses are essential to capture individual molecular profiles and develop tailored countermeasures [91]. Furthermore, the marked heterogeneity observed

in our cohort highlights the growing understanding that genetic variability significantly modulate physiological responses to microgravity, supporting the need for a shift from universal to personalized strategies for astronaut health management during long-duration missions [90, 92].

5. Conclusions

5.1. T cell transcriptomes demonstrate dynamic pathway-specific responses to microgravity

Gene expression profiles and temporal trajectories confirm that T cell transcriptional profiles undergo a dynamic pathway-specific, time-dependent adaptation to microgravity, rather than a static or monotonic shift. Coordinated displacements from background (-7 days) through the SMG period (7–21 days) indicate a shared physiological response that peaks during the middle-to-late exposure phase. The subsequent partial return toward baseline positions by day 28 further supports a recovery trajectory, underscoring that the transcriptomic impact of microgravity is reversible.

5.2. T cell transcriptome modulation by microgravity in vivo affects key immune and oncogenic signaling gene networks

In addition to global expression shifts, the PCA-defined variance points to biologically meaningful perturbations in specific regulatory pathways. The involvement of oncogenic and immunoregulatory cascades is particularly noteworthy, as these pathways are critical for maintaining T-cell homeostasis and functional integrity. This suggests that microgravity does not simply induce general transcriptional noise but rather has targeted effects on molecular pathways linking immunity, cell growth control, and stress-responsive signaling, potentially with implications for host defense and long-term cellular health.

5.3. T cell transcriptome pathway-specific responses to microgravity in vivo are individually variable

Despite the presence of a common temporal signature, the magnitude and direction of these pathway-specific responses are strongly modulated by baseline inter-individual differences. The stratification of the cohort demonstrates that volunteer identity is the dominant source of transcriptional variance. This marked heterogeneity implies that the same microgravity stimulus elicits divergent molecular outcomes across individuals, likely driven by pre-existing baseline transcriptomic profiles. Therefore, studies of T cell adaptation to spaceflight must account for significant interindividual variability, highlighting the need for personalized biomarkers and tailored countermeasures in future space missions.

Supplementary File S6

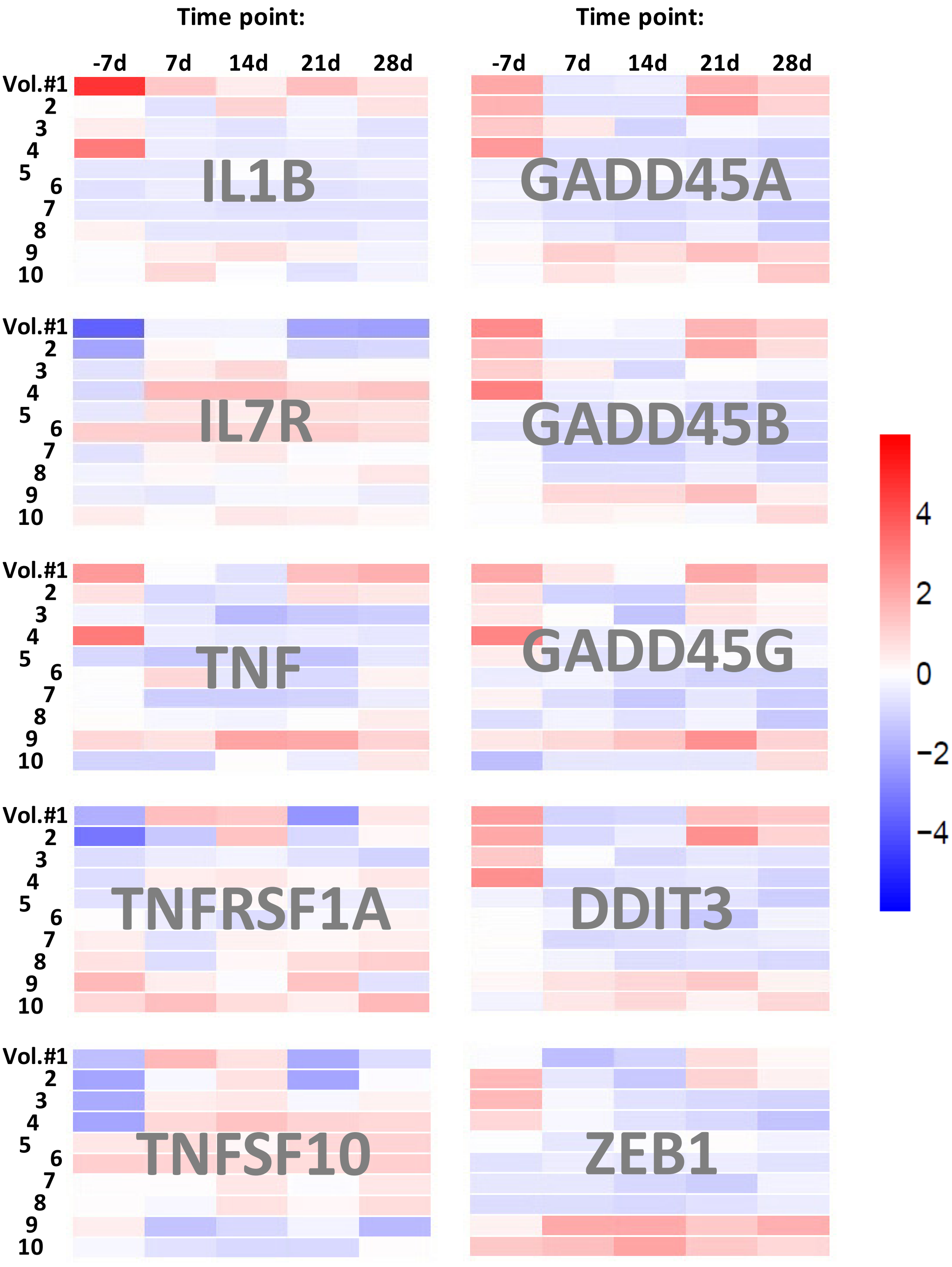

Expression patterns of 10 hub genes distributed across nine of top 12 KEGG pathways across the SMG time course

The figure comprises 10 DEG heatmap panels. Expression values for each of the 10 hub DEGs were analyzed for each of the 10 volunteers across five time points (-7 days baseline, 7, 14, 21 days, and 28 days recovery) and are shown as heatmaps, with volunteer number (Vol. #1-10) on the vertical axis and time points on the horizontal axis.
Color scale indicates log2 fold change, with red indicating upregulation and blue indicating downregulation.

Supplementary File S7

Table S1 RNA-seq data Quality Control summary

Sample name	Raw reads	Clean reads	Raw_data (G)	Clean_data (G)	Error_rate (%)	Q20 (%)	Q30 (%)	GC_content (%)
v1_bg	46796536	45047420	7G	6.8G	0.02	98.23	95.08	51.17
v1_7d	46724140	45443518	7G	6.8G	0.02	98.26	95.05	51.41
v1_14d	49098090	47808234	7.4G	7.2G	0.02	98.26	95.03	51.42
v1_21d	50853356	49543672	7.6G	7.4G	0.02	98.24	95.04	51.15
v1_28d	45631680	44183152	6.8G	6.6G	0.02	98.32	95.20	51.44
V2_bg	44888308	44070814	6.7G	6.6G	0.03	96.96	91.97	50.53
V2_7d	45780324	44950544	6.9G	6.7G	0.02	98.23	94.93	51.43
V2_14d	49696444	48885274	7.5G	7.3G	0.02	98.28	95.03	51.84
V2_21d	48890512	47973484	7.3G	7.2G	0.02	98.24	94.99	51.17
V2_28d	47399960	46252468	7.1G	6.9G	0.02	98.08	94.62	51.27
V3_bg	42573974	41761114	6.4G	6.3G	0.02	98.28	95.08	51.29
V3_7d	46350786	45183730	7G	6.8G	0.02	98.13	94.71	51.42
V3_14d	49702216	48891720	7.5G	7.3G	0.02	98.29	95.09	51.51
V3_21d	48054508	47100934	7.2G	7.1G	0.03	97.85	94.03	52.22
V3_28d	49432398	48650944	7.4G	7.3G	0.02	98.22	94.94	52.22
V4_bg	49481938	48596818	7.4G	7.3G	0.02	98.13	94.71	51.52
V4_7d	47134094	45654534	7.1G	6.8G	0.02	98.20	94.94	51.03
V4_14d	51958240	51008980	7.8G	7.7G	0.03	97.59	93.49	51.90
V4_21d	53697306	52809476	8.1G	7.9G	0.03	97.87	94.13	52.18
V4_28d	49796900	49047826	7.5G	7.4G	0.02	98.26	95.02	51.67
V5_bg	55237482	54768516	8.3G	8.2G	0.02	98.00	94.30	51.17
V5_7d	49361302	48825464	7.4G	7.3G	0.03	97.96	94.17	50.84
V5_14d	48631600	48096792	7.3G	7.2G	0.03	97.90	94.06	51.07
V5_21d	45971544	45540760	6.9G	6.8G	0.03	97.80	93.74	50.90
V5_28d	71499786	70731156	10.7G	10.6G	0.03	97.82	93.80	51.10

Table S1 (continued) RNA-seq data Quality Control summary

Sample name	Raw reads	Clean reads	Raw_data (G)	Clean_data (G)	Error_rate (%)	Q20 (%)	Q30 (%)	GC_content (%)
V6_bg	53495072	52919934	8G	7.9G	0.03	97.88	93.95	51.13
V6_7d	45084894	44596554	6.8G	6.7G	0.02	98.06	94.42	51.10
v6_14d	56037540	55554902	8.4G	8.3G	0.02	98.01	94.28	51.25
V6_21d	51215454	50711418	7.7G	7.6G	0.03	97.82	93.83	51.82
V6_28d	59565236	58929888	8.9G	8.8G	0.03	97.68	93.47	51.03
V7_bg	43780576	43160272	6.6G	6.5G	0.03	97.92	94.07	51.34
V7_7d	54201652	53636880	8.1G	8G	0.02	98.01	94.31	51.09
V7_14d	45768062	45288206	6.9G	6.8G	0.03	97.98	94.23	51.26
V7_21d	44871354	44441408	6.7G	6.7G	0.02	98.04	94.38	51.41
V7_28d	57929396	57350812	8.7G	8.6G	0.03	97.96	94.18	50.74
V8_bg	46252376	45748826	6.9G	6.9G	0.02	98.04	94.38	51.26
V8_7d	50537130	49962768	7.6G	7.5G	0.02	98.02	94.35	51.37
V8_14d	44930164	44472992	6.7G	6.7G	0.03	97.77	94.21	51.49
V8_21d	49597028	49134152	7.4G	7.4G	0.03	97.88	94.27	51.03
V8_28d	53691206	53138572	8.1G	8G	0.03	97.73	94.23	50.84
V9_bg	47871874	47407208	7.2G	7.1G	0.03	97.96	94.17	51.19
V9_7d	40271240	39819262	6G	6G	0.02	98.02	94.31	51.34
V9_14d	41146384	40766406	6.2G	6.1G	0.03	97.77	93.71	50.95
V9_21d	44522088	44035564	6.7G	6.6G	0.03	97.88	93.95	50.79
V9_28d	56435610	55731730	8.5G	8.4G	0.03	97.77	93.56	50.65
V10_bg	44473788	43926756	6.7G	6.6G	0.03	97.77	94.17	51.15
V10_7d	48830432	48333688	7.3G	7.3G	0.03	97.77	93.76	51.61
V10_14d	50709738	50073484	7.6G	7.5G	0.03	97.77	93.42	50.78
V10_21d	47444086	46812424	7.1G	7G	0.03	97.77	93.64	50.69
V10_28d	50713758	50213090	7.6G	7.5G	0.03	97.77	93.05	50.97

Supplementary File S8

Table S2 **List of software used for RNA-seq data analysis**

Analysis	Software	Version	Parameters	Remarks
Mapping	Tophat	V2.0.12	mismatch = 2	mapping to a reference
Quantification	HTSeq	v0.6.1	-m union	
Differential Expression Analysis	DEGseq	v1.12.0	$\lvert \log_2$Fold change$\rvert > 1$; Padj < 0.005	DEGSeq used for samples without bioreplicates.<br><br>EdgeR used for specific conditions.
	EdgeR	3.0.8	Padj < 0.05	
GO Enrichment	GOSeq, topGO, hmmscan	V2.12	Corrected P-Value<0.05	hmmscan
KEGG Enrichment	KOBAS	v3.0	Corrected P-Value<0.05	

Table S3 **Differential Gene Expression Analysis**

Type of Analysis	Software	Normalization	p-value estimation model	FDR estimation method
Differential Expression Analysis without bioreplicates	DEGseq	TMM	$\lvert log_2$Fold change$\rvert > 1$; Padj < 0.005	BH
Comment	The readcount value of the ith gene in the jth sample is K_{ij}, then<br>Negative binomial distribution: K_{ij} NB(μ_{ij},σ_{ij}2)<br>Poisson distribution: K_{ij} P(μ_{ij})			
